# Redox mediator-enhanced charge storage in dimensionally tailored nanostructures towards flexible hybrid solid-state supercapacitors†

**DOI:** 10.1039/d3na00279a

**Published:** 2023-08-09

**Authors:** Ritik Mohanty, Kaushik Parida, Kulamani Parida

**Affiliations:** a Centre for Nanoscience and Nanotechnology, Siksha 'O'Anusandhan (Deemed to be University) Bhubaneswar 751030 Odisha India kulamaniparida@soa.ac.in ritikphy.cet@gmail.com; b Department of Polymer & Process Engineering, Indian Institute of Technology Roorkee Saharanpur Campus Uttarakhand 247001 India kaushik.parida@pe.iitr.ac.in

## Abstract

Although extensive research has been performed on metal oxide-based supercapacitors during recent years, they remain lacking in their intrinsic conductivity and stability. To resolve this, 1D/2D heterostructure materials are being utilized, which significantly improves the performance and stability of both materials while employing their synergistic advantage consisting of morphologically tuned surfaces and superior electroactive sites. However, the performance remains unsatisfactory due to the sluggish faradaic reaction at the electrode/electrolyte interface. To address this challenge, we combined the synergistic advantage of morphological nanoengineering and the fast reaction kinetics of redox mediators, thus anticipating superior energy storage performance. A novel 1D/2D heterostructure of ZnCo_2_O_4_ (ZCO) and GaN was designed and implemented for the first time, and it demonstrated an excellent specific capacitance of 1693 F g^−1^ in the mixed electrolyte of KOH and K_4_[Fe(CN)_6_]. The all-solid-state flexible hybrid supercapacitor delivered an energy density of 92.63 W h kg^−1^ at a power density of 1287.52 W kg^−1^, with superb stability and mechanical endurance that outperformed previously reported ZCO-based materials. Additionally, we delineated the underlying mechanism governing the utilization of redox mediators along with morphological nanoengineering, which will facilitate the current development of state-of-the-art energy storage systems.

## Abstract 2

Redox mediator-amplified charge storage in a dimensionally engineered 2D GaN@1D ZnCo_2_O_4_ nanostructure towards high-performance flexible supercapacitors.

## Graphical abstract

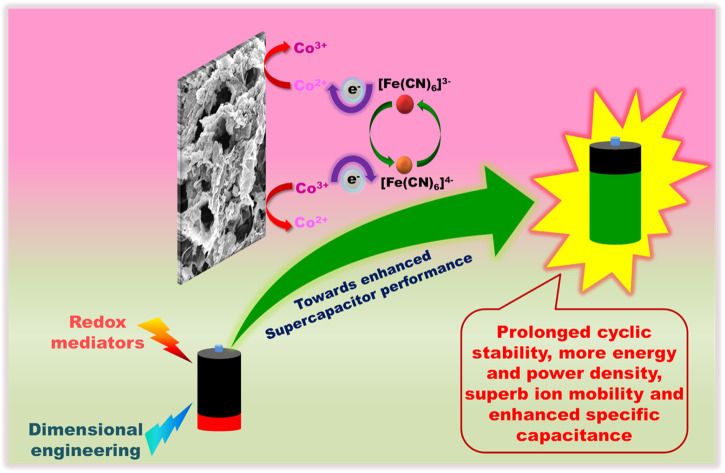

## 1. Introduction

With the progress in science and technology, energy storage devices are currently playing a vital part in our day-to-day life. Among all the electrochemical storage devices, supercapacitors have emerged as promising energy storage devices because of their adequate energy and power delivery coupled with ultra-high cycle life. As per the charge storage mechanism, electrochemical capacitors have been classified as electrical double-layer capacitors (EDLCs), which arise from ion adsorption, and pseudo capacitors, which originate from surface faradaic redox reactions.^1–6^ As conventional carbon-based electrode materials have approached the theoretical capacity limits, carbon-free alternatives with ultra-high capacitance are urgently required.^7,8^

Alternatively, transition metal oxides, with their high charge storage capacities based on the conversion reaction mechanism as well as their abundance, have been extensively utilized. Considering the example of ternary metal oxide ZnCo_2_O_4_ (ZCO), two different metal cations display superior redox reactions that amplify the overall electrochemical performance.^9^ Although its theoretical capacity is high, it is accompanied by low conductivity and sluggish chemical kinetics. Metal nitrides are suitable electrode materials for superior and durable energy storage devices because of their excellent electrical conductivity and high specific capacitance.^10,11^ However, the exploration of compounds of group III–V materials as electrodes for supercapacitor devices is still in its infancy.

Gallium nitride (GaN), a group III–V direct bandgap semiconductor possessing a wurtzite crystal structure, has been widely explored for various stable and long-term optoelectronic and sensing applications due to its high voltage capability and melting point.^11–13^ In addition, it exhibits superior thermal conductivity, extra charge carriers per volume, excellent electron velocities, and chemical and physical stability, which are the desired traits of a satisfactory electrode material for supercapacitor application. Thus, ZnCo_2_O_4_–GaN (G-ZCO) nanocomposites could be an ideal candidate for durable high-performance energy storage devices.

Additionally, the use of redox mediators to boost the performance of supercapacitors has emerged as an effective and sustainable strategy. A typical redox mediator can dissolve into an existing electrolyte system, which is a safe, sustainable, and reliable operational approach. Additionally, the redox reaction mechanism occurs at the electrode surface, and is reversible.^14^ The inclusion of redox mediators acts as an auxiliary electron buffer source to compensate for the moderate nature of the faradaic reaction. Thus far, various redox mediators such as potassium iodide, potassium ferrocyanide, and hydroquinone have been used with aqueous electrolytes to amplify the energy density of the carbonaceous material-based supercapacitors.^15,16^

There have been only a few investigations on pseudocapacitive materials whereby enhancement of the overall energy storage activity occurs due to the addition of some redox active species. In this case, the enhanced activity can be attributed to the improved ionic conductivity along with the available number of additional redox pairs. Thus, it is an enforceable and sustainable method to augment the electrochemical activity through the synergistic effect of a hybrid material with an organized interconnected nanostructure that will assure the unhindered access of electrolyte ions to more possible surfaces and shorten the transport path of ions and electrons. Further incorporation of a redox mediator can deliver additional pseudocapacitance originating from near-surface reversible redox reactions to compensate for slow redox reactions. An additional advantage such as inhibition of current collector corrosion has been allied with the use of redox mediators.

To the best of our knowledge, there is no such evidence available on the effect of redox mediator-induced charge storage in novel 1D/2D heterostructures, *i.e.*, G-ZCO for asymmetric flexible supercapacitor applications. In the current study, G-ZCO was fabricated by a hydrothermal method, followed by a post-calcination route. G-ZCO exhibited superior electrochemical activity due to the nanoscale morphological engineering along with the presence of redox mediators such as potassium ferrocyanide (*i.e.*, K_4_[Fe(CN)_6_], denoted as KFeCN) is the aqueous electrolyte, which will provide additional redox pairs for augmenting the energy storage activity.

Our findings strongly prove that facile shuttling of Fe(CN)_6_^3−^/Fe(CN)_6_^4−^ synergistically facilitates the electron loss and gain of Co ions, as well as providing an enormous contribution to the pseudocapacitance of the as-prepared dimensionally tailored nanohybrid. Our designed compound displayed an outstanding specific capacitance of 1693 F g^−1^ at a sweep rate of 5 mV s^−1^ in the mixed aqueous electrolyte of 2 M KOH + 0.1 M KFeCN with a 98% coulombic efficiency and long-term stability of 93% capacitance retention after 6000 continuous charge–discharge cycles. The as-fabricated ultra-flexible all-solid-state gel electrolyte-based asymmetric hybrid supercapacitor device exhibited the specific capacitance of 176 F g^−1^ in 1 A g^−1^ using G-ZCO as the positive electrode and activated fullerene (A-C_60_) as the negative electrode.

Here, A-C_60_ was adopted in the flexible asymmetric supercapacitor device (FASD) because of its impressive mechanical and electrochemical properties along with superb stability while undergoing extreme mechanical deformations. The FASD delivered an energy density of 92.63 W h kg^−1^ at a power density of 1287.52 W kg^−1^, with 96.7% stability up to 10 000 cycles at a moderate current density of 6 A g^−1^ when subjected to various bending and twisting tests.

## 2. Materials and methods

All the chemicals used in our work were of analytical grade and were purchased from Sigma-Aldrich, Alfa Aeser, HiMedia, and Merck; they were used as received without any further refinement. Zinc nitrate [Zn(NO_3_)_2_·6H_2_O], cobalt nitrate [Co(NO_3_)_2_·6H_2_O], ammonium fluoride [NH_4_F], urea [CO(NH_2_)_2_], gallium nitride [GaN] nano-powder, fullerene [C_60_] powder, potassium ferrocyanide [K_4_[Fe(CN)_6_]], potassium hydroxide [KOH], anhydrous ethanol, carbon black, and Nafion were purchased, and de-ionized (DI) water was used for all experiments.

### 2.1 Synthesis of zinc cobaltite (ZCO)

A simple hydrothermal method followed by an calcination technique was chosen for the synthesis of the ZnCo_2_O_4_ nanorods. We dissolved 1 mM Zn(NO_3_)_2_·6H_2_O, 2 mM Co(NO_3_)_2_·6H_2_O, 2 mM NH_4_F, and 5 mM CO(NH_2_)_2_ in 40 mL of DI water *via* continuous stirring until a clear solution formed, which was transferred into a 100 mL Teflon-lined stainless steel autoclave that was heated in a hot air oven at 125 °C for 5 h. The resulting precipitate was collected and washed thoroughly several times with DI water and ethanol by centrifugation and vacuum drying. Finally, the powder was annealed in a muffle furnace in an ambient atmosphere at 400 °C for 2 h at a heating rate of 2° min^−1^.

### 2.2 Preparation of gallium nitride-incorporated zinc cobaltite (G-ZCO)

G-ZCO was prepared considering the identical hydrothermal procedure as mentioned above. A fixed amount (5 wt% or 38.37 mg) of GaN powder was added to an aqueous solution containing the Zn and Co nitrate salt precursor, and then stirred and sonicated until the formation of a clear solution. Thereafter, the solution was transferred to an autoclave and heated at 125 °C for 5 h. The resulting precipitate was collected and washed with DI water and ethanol, and then vacuum dried. Finally, the dried powder was annealed at 400 °C for 2 h in an ambient atmosphere with a heating rate of 2° min^−1^. For the controlled preparation, we loaded different weight percentages of GaN in ZCO (1, 3, 5, and 10 wt%), and among all 5 wt% loaded sample displayed extraordinary supercapacitive activity. Therefore, GaN loading of 5 wt% in ZCO was considered an ideal composite for further physicochemical and electrochemical studies, and is abbreviated as G-ZCO.

### 2.3 Preparation of activated fullerene (A-C_60_) for the negative electrode

Activation of commercial fullerene was performed by adopting the methods from our previous reports,^9^ where KOH was used as a chemical activator. Initially, 100 mg C_60_ powder and 400 mg KOH were thoroughly ground until a uniform powder-like texture was attained. Thereafter, thermal treatment was performed at 600 °C, with a ramping rate of 5 °C min^−1^ under an inert environment. The obatained compounds were then cooled, thoroughly washed with distilled water until the supernatant attained a pH of 7, and vacuum-dried overnight at 60 °C.

### 2.4 Preparation of the gel electrolyte for the FASD

Redox-active polymer gel electrolyte was prepared *via* a solution casting method by dissolving 1 g of PVA, 1.1 g of KOH, and 0.26 g of KFeCN in DI water under vigorous stirring at 75 °C for 5 h until complete dissolution. Thereafter, it was stirred at a moderate rate until a homogenous viscous paste formed at the bottom of the beaker. Then, it was solution-casted on a glass slide to form a uniform structure, and the excess water was evaporated by drying at 60 °C inside a vacuum oven. After drying, the polymer gel electrolyte was removed from the glass slides and cut into the desired shapes for use in all-solid-state supercapacitor devices.

## 3. Results and discussion

### 3.1 Formation mechanism

**Scheme 1:**
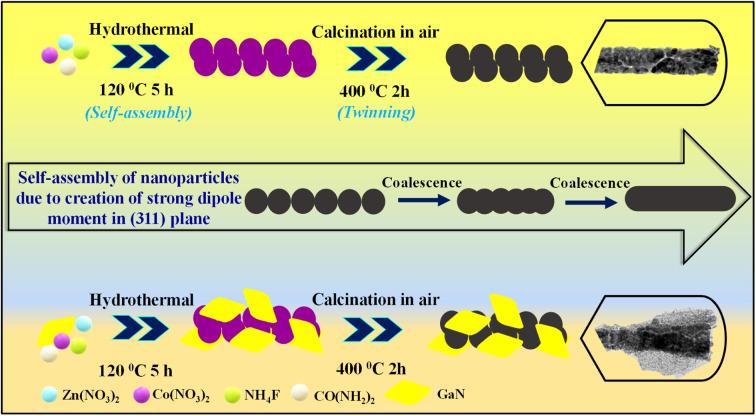
Formation mechanism displaying the synthesis scheme.

The possible formation mechanism is shown in Scheme 1, where an example of crystal seed growth in the crystal nucleus solution is taken into consideration, and nanoparticles aggregate and their self-assemblies initiate the 1D rod-like morphology. In the primary stage of the reaction due to the hydrothermal process, NH_4_F and urea act as a structure director as well as a complexing agent respectively. Metal source precursors such as Zn^2+^ and Co^2+^ react with urea, and many nanoparticles were formed and gathered by lowering their interfacial energy, which will initiate crystal nucleation and the growth process. Because of the permanent dipole moment in the nanoparticles, the formation of nanoparticles was induced, and they were oriented into a long linear chain in the solution.

In the present study, the (311) plane of ZCO was polar because of the opposite layer formation by Zn^2+^ and Co^2+^, which created a strong dipole moment in the (311) plane so that the ZCO nanoparticles oriented to the (311) plane attachment to form nanorods. After subsequent calcining at 400 °C, the prepared precursor was fully converted to ZCO by maintaining the inner and outer morphology. Furthermore, because of the breakdown of some organic species at higher temperatures, the calcination process amplified the porosity and crystallinity. Lastly, by introducing 2D GaN into the ZCO materials matrix, the conductivity, porosity, and crystallinity were improved, and thus, the 1D/2D hybrid was able to deliver the extra-ordinary supercapacitive performance.

### 3.2 Structural and surface chemical analysis

**Fig. 1:**
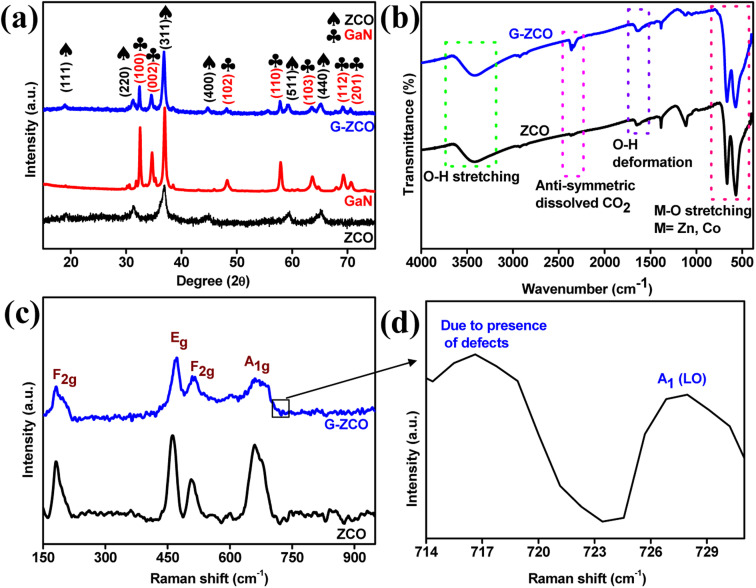
(a) XRD patterns for ZCO, GaN, and G-ZCO. (b) FTIR and (c and d) Raman spectra of ZCO and G-ZCO.

Fig. 1a displays the X-ray diffraction spectrum for ZCO, GaN, and G-ZCO, where the peaks at 2*θ* values of 19.06°, 31.40°, 36.94°, 45.07°, 59.44°, and 65.22° are well indexed with the (111), (220), (311), (400), (511), and (440) crystal planes of spinel ZCO with JCPDS No. 23-1390, respectively.^9,17^ As displayed in Fig. 1a, the diffraction angles and interplanar spacings for GaN are consistent with the standard JCPDS card No. 50-0792.^18^

In the G-ZCO nanocomposite, both phases are evenly found, which was attributed to the proper composite formation. The well-recognized peaks of G-ZCO at 2*θ* values of 18.87°, 31.21°, 32.46°, 34.49°, 36.83°, 44.74°, 48.02°, 57.87°, 59.27°, 63.49°, 65.21°, 69.12°, and 70.52° were well indexed with the (111), (220), (100), (002), (311), (400), (102), (110), (511), (103), (440), (112), and (201) crystal planes. This shows all the diffraction peaks for GaN and ZCO with a minute lower 2*θ* angle shifting, indicating the presence of compressive stress along with an increase in the inter-planar spacing of G-ZCO.^19^ The increase in spacing between the planes favors rapid ion migration inside the crystal structure and is demonstrated by extraordinary supercapacitive properties. There were no single impurity peaks of individual Ga, Zn, Co oxides, or hydroxides, suggesting the high phase purity of the material.

Fourier transform infrared (FT-IR) spectroscopy analysis was conducted to gain more insight into the chemical structure of the as-prepared materials. The absorption peaks in the FT-IR spectra of ZCO and G-ZCO are displayed in Fig. 1b. The broad distinct IR absorption peak centered at 570 cm^−1^ for the neat GaN sample is attributed to the A_1_ and E_1_ transverse optical lattice phonon modes of GaN (Fig. S1†).^11,20^ Furthermore, for the ZCO and G-ZCO samples, the peak centered near 560 and 660 cm^−1^ was associated with the spinel structure and F_1u_ vibration modes. Apart from this, all the analysed samples possessed neat peaks at 2360 and 3450 cm^−1^, which were due to the anti-symmetric dissolved CO_2_ and O–H stretching.^21^

There were no distinct observable peaks of GaN in the G-ZCO due to the dominant nature of the ZCO and low loading amount of GaN. Rather, a positive shift in the wavelength as well as a reduction in peak height was observed. There were no noticeable peaks of Ga–O centered at 480 or 650 cm^−1^, which strongly signifies the phase purity and chemical stability of the GaN in G-ZCO.

Confocal Raman spectroscopic analysis was also performed to gain additional insight into the chemical bonding and structures of the as-prepared materials. The Raman spectra curves displayed in Fig. 1c and d show the presence of defects along with modes of vibrations associated with the synthesized samples. The neat ZCO displays characteristic peak signals at approximately 181 cm^−1^, 462 cm^−1^, 507 cm^−1^, and 660 cm^−1^. By contrast, in G-ZCO, a positive Raman shift occurred, where the characteristics peaks well indexed at approximately 182 cm^−1^, 471 cm^−1^, 512 cm^−1^, 662 cm^−1^, 716 cm^−1^, and 727 cm^−1^ were attributed to the F_2g_, E_g_, F_2g_, and A_1g_ modes for spinel ZCO, along with the presence of defects and the A_1_(LO) longitudinal modes of GaN, respectively.^22,23^

X-ray photoelectron spectroscopy (XPS) analysis was performed to evaluate the elemental composition along with the surface chemical valence of ZCO and G-ZCO. Fig. S2† shows the XPS survey spectra of the as-prepared ZCO and G-ZCO, which confirms the presence of elemental Zn, Co, O, C, Ga, and N in the sample, and a slight negative shift of binding energy was observed for the G-ZCO sample as compared with ZCO. The lower energy shift validated the heterostructure formation due to the incorporation of electron-rich GaN into the ZCO framework.

**Fig. 2:**
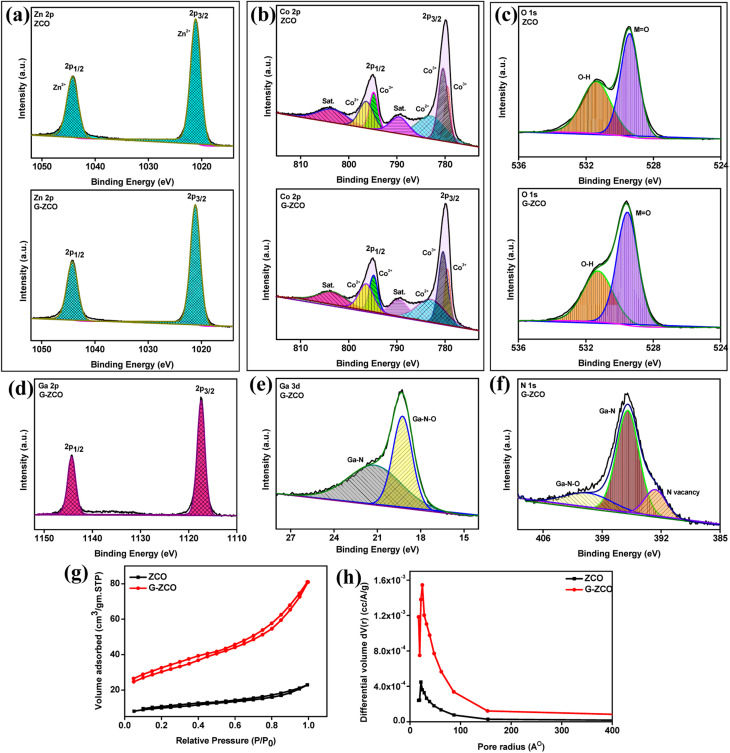
High-resolution deconvoluted XPS analysis of (a) Zn, (b) Co, and (c) O of ZCO and G-ZCO, and (d) Ga 2p, (e) Ga 3d, and (f) N 1s of G-ZCO. (g) Isotherm plots and the (h) corresponding pore size distribution for ZCO and G-ZCO.

For further analysis, the high-energy XPS spectra were deconvoluted and are shown in Fig. 2. The deconvoluted Zn 2p spectra shown in Fig. 2a illustrate two intense peaks corresponding to the Zn 2p_3/2_ and 2p_1/2_ dual spin–orbit doublets associated with the Zn^2+^ oxidation state. In neat ZCO, the Zn 2p_3/2_ and Zn 2p_1/2_ states are located at approximately 1044.23 and 1021.12 eV, respectively. Likewise, in G-ZCO, Zn 2p_3/2_ and Zn 2p_1/2_ are centered at approximately 1044.02 and 1020.93 eV, respectively, which strongly clarifies that Zn^2+^ in the normal state was present in the as-prepared nanocomposite.

Furthermore, a spin-splitting value of approximately 23.13 eV (for ZCO) and 23.14 eV (for G-ZCO) was calculated, which again confirms the presence of Zn atoms in a 2+ oxidation state.^24^ The deconvoluted spectra of Co 2p are presented in Fig. 2b, which clarifies that the as-prepared materials correspond to the 2+ and 3+ oxidation states associated with the Co 2p_3/2_ and 2p_1/2_ states. The Co 2p peaks in neat ZCO were indexed at approximately 780.42, 782.93, and 796.37 eV for Co^2+^, and 779.52 and 794.83 eV for Co^3+^ states, with the presence of two satellite peaks at 789.49 and 803.34 eV. Likewise, Co 2p peaks for G-ZCO were observed at approximately 780.22, 782.73, and 796.17 eV for Co^2+^, and 779.32 and 794.63 eV for Co^3+^ states.

Because of the rapid change in the coulombic potential, two satellite peaks appeared at approximately 789.16 eV and 803.142 eV. The spin energy variance between Co 2p_3/2_ and 2p_1/2_ for both samples was calculated to be approximately 15.18 eV and 15.14 eV for ZCO and G-ZCO, respectively,^25–27^ which is in accordance with values from previous studies by strongly authenticating the co-existence of the 2+ and 3+ oxidation states, among the 2+ state being predominant. With the diverse oxidation states and high valency of Co ions, enhanced supercapacitive activity can be expected with the ability to tune the sluggish chemical kinetics.

The detailed spectrum (Fig. 2c) of O 1s features 2 photoelectron peaks for the ZCO and G-ZCO samples. There were two peaks for neat ZCO at approximately 531.38 eV and 529.40 eV due to O–H and M=O, respectively, and similar results were also obtained for G-ZCO. The peak at approximately 531.06 eV was attributed to the O–H caused by the oxygen defects/vacancies of the chemo-adsorbed water molecule or by the environmental trapped oxygen, and another peak located at approximately 529.33 eV signified metal–oxygen bonding (M=O).^9,27^

After the successful formation of the G-ZCO nanostructure, it was found that Ga was present in the Ga 3d and 2p states. The detailed XPS spectrum of Ga 2p for the G-ZCO sample displayed two peaks for Ga 2p_1/2_ and Ga 2p_3/2_ corresponding to the binding energy of approximately 1144.1 and 1117.22 eV, respectively (Fig. 2d). A spin-splitting value of approximately 26.82 eV for Ga 2p was observed for G-ZCO, which was in accordance with the reported literature. Furthermore, the deconvoluted Ga 3d spectrum is presented in Fig. 2e, which displays peaks at approximately 19.06 eV (Ga–N–O) and 20.98 eV (Ga–N). In Fig. 2f, N 1s features 3 photoelectron peaks centered at approximately 395.76, 400.45, and 392.48 eV due to the Ga–N, Ga–N–O, and N defects, respectively.^11,28,29^ The obtained results are consistent with X-ray diffraction (XRD), FT-IR, and Raman analyses by further confirming the successful incorporation of GaN in ZCO. For compressive interpretation, the associated XPS data for the as-synthesized samples have been summarized in Table 1.

**Table 1:** Tabular XPS interpretation of ZCO and G-ZCO

Elements		Binding energy							Ref.
Zn 2p	ZCO	1021.12 eV	1044.23 eV						24
	G-ZCO	1020.93 eV	1044.02 eV						
	Rationale	Zn 2p_1/2_ (Zn 2^+^)	Zn 2p_3/2_ (Zn 2^+^)						
	Spin-splitting value (*Δ*)	*Δ* = 23.13 eV (for ZCO) and 23.14 eV (for G-ZCO)							
Co 2p	ZCO	779.52 eV	794.83 eV	780.42 eV	782.93 eV	796.37 eV	789.49 eV	803.34 eV	25–27
	G-ZCO	779.32 eV	794.63 eV	780.22 eV	782.73 eV	796.17 eV	789.16 eV	803.14 eV	
	Rationale	Co 2p_3/2_ (Co 3^+^)	Co 2p_1/2_ (Co 3^+^)	Co 2p_3/2_ (Co 2^+^)	Co 2p_3/2_ (Co 2^+^)	Co 2p_1/2_ (Co 2^+^)	Satellite peak of Co 2p_3/2_	Satellite peak of Co 2p_1/2_	
	Spin-splitting value (*Δ*)	*Δ* = 15.18 eV (for ZCO) and 15.14 eV (for G-ZCO)							
O 1s	ZCO	531.38 eV	529.40 eV						9 and 27
	G-ZCO	531.06 eV	529.33 eV						
	Rationale	O–H	M=O						
N 1s	G-ZCO	395.76 eV	400.45 eV	392.48 eV					11, 28 and 29
	Rationale	Ga–N	Ga–N–O	N defects					
Ga 2p	G-ZCO	1117.22 eV	1144.1 eV						
	Rationale	Ga 2p_3/2_	Ga 2p_1/2_						
	Spin-splitting value (*Δ*)	*Δ* = 26.82 eV (for ZCO)							
Ga 3d	G-ZCO	19.06 eV	20.98 eV						
	Rationale	Ga–N–O	Ga–N						

The specific surface area and pore analysis of the as-prepared ZCO and G-ZCO were studied by N_2_ adsorption and desorption isotherms. From Fig. 2g, it can be inferred that both samples followed the type-IV isotherms constituting mainly mesopores.^9^ The Brunauer–Emmett–Teller (BET) surface area of ZCO and G-ZCO was calculated to be 35.59 and 107.85 m^2^ g^−1^, respectively, *via* multi-point analysis. The available specific surface area in G-ZCO was higher due to the formation of heterostructures with GaN, which offers a large number of accessible electroactive pores for improved supercapacitive properties.

The pore size distribution for both samples shown in Fig. 2h indicates the available fine mesoporous texture, where the average pore radius of ZCO and G-ZCO was 19.92 and 23.21 Å, respectively. Because of the large pore radius in G-ZCO, it can accumulate huge amounts of electrolyte ions inside its void and perform rapid diffusion to interact with the core of the electrode surface. The availability of additional accessible pores in G-ZCO permits sufficient surface redox reaction so that high capacitance from the faradaic redox reaction can be offered.

### 3.3 Morphological analysis

**Fig. 3:**
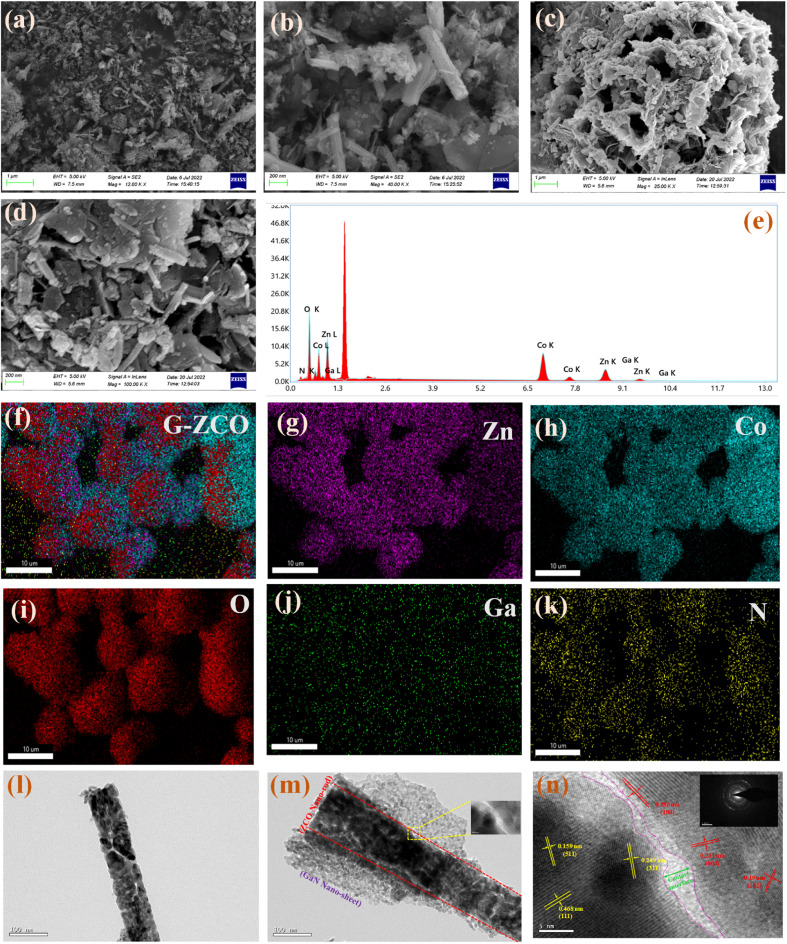
FESEM images of (a and b) ZCO and (c and d) G-ZCO. (e) EDX spectra and (f–k) color elemental mapping of as-prepared G-ZCO. TEM micrographs of (l) ZCO and (m) G-ZCO. (n) HRTEM image, with the inset showing the SAED pattern of G-ZCO.

The outer surface morphology of our synthesized samples was inspected by scanning electron microscopy (SEM). In Fig. 3a and b, non-uniform nanorods were observed for ZCO that were primarily constructed by the agglomeration of nanoparticles. The surface morphology of G-ZCO shown in Fig. 3c and d further clarifies the proper anchoring of the GaN sheets onto the ZCO rods, which created a 3D hollow spherical flower-like structure constructed by uniform agglomeration of 1D ZCO nanorods and 2D GaN. The as-constructed 3D structure possesses a more specific surface area along with a greater number of available electroactive sites as compared to pristine ZCO, which will be beneficial for the facile transport of ions. Furthermore, the hollow flower-like structure will also provide a rapid in-and-out path for the diffused ions from the aqueous electrolyte solution.

The corresponding energy dispersive X-ray spectroscopy and elemental mapping results for ZCO (Fig. S3†) and G-ZCO (Fig. 3e–k) are shown. The proper heterostructure formation between ZCO and GaN appears in Fig. 3e, where the color mapping confirms that elemental Zn, Co, O, Ga, and N are evenly distributed throughout the G-ZCO sample. Because of these fascinating morphological properties, we can anticipate enhanced supercapacitive activity in G-ZCO.

The interior subtle structure of ZCO and G-ZCO was investigated by transmission electron microscopy (TEM). As displayed in Fig. 3l, ZCO possesses a rod-like shape due to the primary agglomeration of nanoparticles. The high resolution (HR)-TEM image of ZCO in Fig. S4† shows an active crystal facet and a selected area electron diffraction (SAED) pattern with an amorphous blurry halo that confirms its phase purity, decreased crystallinity, and polycrystalline nature, which is in accordance with the XRD data. Furthermore, the TEM image of G-ZCO in Fig. 3m shows tightly anchored GaN nanosheets stacked over ZCO rods without any agglomeration, which is in agreement with the morphological analysis *via* field emission (FE)-SEM.

For the G-ZCO sample, the well-resolved lattice spacing of approximately 0.249, 0.468, and 0.159 nm (denoted in yellow) can be indexed to the (311), (111), and (511) crystal facets of ZCO, respectively, while the 0.286, 0.261, and 0.19 nm lattice spacing (denoted in red) corresponds to the (100), (002), and (102) crystal planes of GaN, respectively (Fig. 3n).^17–19^ The inset of Fig. 3n exhibits the SAED pattern of G-ZCO, where ill-defined concentric diffraction rings along with a decrease in intensity of an amorphous blurry halo are observed (as compared to ZCO) that confirm its increase in crystallinity.

Notably, the distinct and long-range contact interface dramatically enhances the electronic structure inside the materials for amplification of the electronic enrichment. Thus, there is an enormous amount of abundant active sites in the G-ZCO nanohybrid for redox reaction, due to the ability of 1D ZCO and 2D GaN to amplify the sluggish faradaic kinetics *via* their synergistic effect. Lastly, there is great promise for G-ZCO in electrochemical energy storage applications because of its abundant electroactive sites, interfaces, pores, and redox-active centers.

### 3.4 Three-electrode electrochemical analysis

The electrochemical performance of all the samples was evaluated in a three-electrode cell, with Pt wire and Hg/HgO acting as the counter and reference electrode, respectively. The working electrode was fabricated with Ni foam as the current collector (Fig. S5†), and various active materials were drop-casted onto it. The detailed procedure for electrochemical characterization and formulas for performance estimation have been mentioned in the ESI.†

Because of the sluggish kinetics in aqueous electrolytes, few studies have been conducted that combined the aqueous solution of K_4_[Fe(CN)_6_] (KFeCN) with aqueous electrolyte systems to augment the electrochemical activity. The enhanced electrochemical performance is attributed to the redox mediator of [Fe(CN)_6_]^4−^/[Fe(CN)_6_]^3−^, which acts as an extra electron buffer source to quickly perform the surface redox reaction. [Fe(CN)_6_]^4−^/[Fe(CN)_6_]^3−^ possesses a Bohr radius of approximately 0.4 nm, and^30,31^ the pore radius of G-ZCO is an average of 2.3 nm, which can accumulate a substantial amount of redox ions to increase the electrochemical kinetics.

Because varying the KFeCN concentration can have an impact on several aspects of supercapacitor performance, including specific capacitance and stability, we varied the KFeCN concentration (0.05 M, 0.1 M, 0.2 M, 0.5 M, 1 M, and 2 M) and then conducted electrochemical measurements to systematically investigate the effect. The experimental results provided strong metrics to evaluate the performance across a range of concentrations and provide a comprehensive understanding of the impact of KFeCN concentration on the supercapacitive behaviour of G-ZCO. For purposes of comparison, the cyclic voltammetry (CV) curves are provided (Fig. S6a†), with a detailed explanation in the ESI text.†

With the gradual increase in the percentage of KFeCN in the KOH, there was also an appreciable increase in the area of the cyclic voltammogram along with a higher current value. Compared to one with any redox mediator, the presence of KFeCN in any concentration beyond 0.5 M redox additive resulted in a larger enclosed area. Furthermore, increasing the redox additive amount to 2 M resulted in no noticeable change in the enclosed area of the CV curve. However, much greater capacitance loss was observed after 1000 cycles (Fig. S6b†), as well as poor rate capability. Hence, 0.1 M KFeCN was chosen as the most optimum concentration for further electrochemical analysis and measurements by considering its balanced traits, such as offering high specific capacitance as well as stability. Thus, in this regard, an aqueous mixture of 2 M KOH and 0.1 M KFeCN was used as the electrolyte in a three-electrode setup for all the forthcoming electrochemical analyses.

**Fig. 4:**
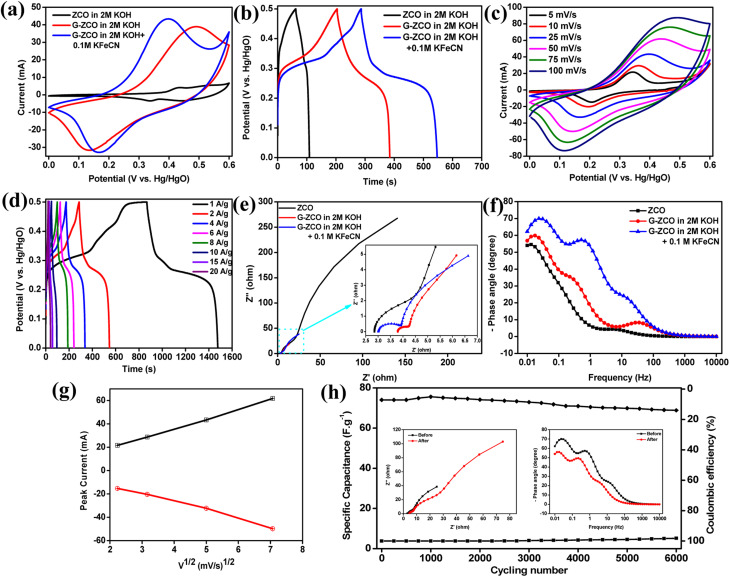
Electrochemical characterization of the as-prepared materials in a 3-electrode setup. (a) CV curves at 25 mV s^−1^ and (b) GCD graphs at 2 A g^−1^ of ZCO and G-ZCO in 2 M KOH and 2 M KOH + 0.1 M KfeCN. (c) CV curves of G-ZCO in 2 M KOH + 0.1 M KFeCN at diverse scan rates. (d) GCD curves of G-ZCO in 2 M KOH + 0.1 M KFeCN at varied current density. (e) EIS curves (Nyquist plots) of ZCO and G-ZCO in 2 M KOH and 2 M KOH + 0.1 M KFeCN (the inset shows a magnified version). (f) Bode plot of ZCO and G-ZCO in 2 M KOH and 2 M KOH + 0.1 M KFeCN. (g) RS plot and (h) cyclic stability and coulombic efficiency at a current density of 10 A g^−1^ for G-ZCO in 2 M KOH + 0.1 M KFeCN (the inset displays before and after Nyquist and Bode plots).

CV and galvanostatic charge/discharge (GCD) curves were carried out on the ZCO and G-ZCO electrodes in 2 M KOH and 2 M KOH + 0.1 M KFeCN electrolytes, and are displayed in Fig. 4a and b. Excitingly, the CV area and GCD curve of the dimensionally engineered G-ZCO are larger than those of the pristine ZCO, which can be attributed to the enhanced hydrophilicity and conductivity, larger surface area, and a greater number of electroactive sites. The more visible enclosed area (Fig. 4a) of G-ZCO in mixed electrolyte rather than neat KOH indicates a significant increase in electrochemical activity after the addition of KFeCN. The more optimal electrochemical performance is attributed to the additional capacitive contribution from the use of a redox mediator, which undergoes a reversible reaction from [Fe(CN)_6_]^3−^/[Fe(CN)_6_]^4−^ to augment the supercapacitive properties. Furthermore, the enhancement in the superb electrochemical activity of G-ZCO is evident from the elongated discharge time displayed in Fig. 4b.

As shown, one can conclude that the synergistic contribution of G-ZCO (from the dimensional engineering) and KFeCN (by the incorporation of redox mediator) is prolongation of the charge–discharge time, along with satisfactory reversibility at higher current densities. Fig. 4c and d represent the CV and GCD curves of G-ZCO, respectively, in mixed electrolytes of KOH and KFeCN, where we can confirm that the as-prepared material displays a pseudo-capacitive nature by examining two distinct redox peaks in the CV curves and a distinct plateau-like GCD graph. The adopted potential range for GCD was slightly lower than the potential used for CV because at a higher potential window, the electrode might fail to charge due to some slow and irreversible reaction of G-ZCO. Thus, we adopted 0 to 0.5 V (potential *vs.* Hg/HgO) as the potential window for GCD curves, which is smaller than that of CV.

The redox peaks in the represented CV curves are mainly ascribed to the reversible redox reactions (especially faradaic) associated with M–O/M–O–OH (M = Zn/Co), which enable the pseudo-capacitive behavior of the as-synthesized hybrids. This above-stated explanation is further supported by eqn (1)–(3):^32,33^

1 ZnCo_2_O_4_ + OH^−^ + H_2_O ↔ ZnOOH + 2CoOOH + e^−^

2 CoOOH + O ↔ CoO_2_ + H_2_O + e^−^

3 Co_2_O_4_^2−^ + OH^−^ + 2H_2_O ↔ 2CoOOH + e^−^

The as-prepared G-ZCO shows the specific capacitance of 1366 F g^−1^ at a current density of 1 A g^−1^ (from GCD curves) along with a gravimetric specific capacitance of 1693 F g^−1^ at a sweep rate of 5 mV s^−1^ (obtained from the CV curves). The poor coulombic efficiency observed at 1 A g^−1^ current density was not suitable for consideration as the highest capacitance of the active material. Hence, we adopted the appropriate lowest current density of 2 A g^−1^ for the capacitance calculation, which displayed the specific capacitance of 476 F g^−1^. There are plentiful channels and pores for ion storage in the dimensionally engineered 1D/2D heterostructure, and thus, the as-synthesized G-ZCO delivered supreme capacity as compared to ZCO (obtained specific capacitance of 361 F g^−1^ at 1 A g^−1^) or G-ZCO (obtained specific capacitance of 764 F g^−1^ at 1 A g^−1^) in pure KOH electrolyte.

The improved specific capacitance was extracted from the synergistically interplayed outcome, *i.e.*, the K^+^ and OH^−^ storage in the interconnected region between ZCO and GaN in the G-ZCO heterostructure, and along with faster shuttling of electrons (due to the use of redox mediators), improved the surface redox reaction, which also dramatically improved the specific capacitances. The comparative specific capacitance concerning scan rate and current density is shown in Fig. S7,† which is as expected. The increase in the scan rate resulted in lower specific capacities due to the increase in internal resistances. The GCD curves also provide insight into the anticipated behavior by their slight decrease in specific capacitance in higher applied current densities due to fractional ion migration into the core of the materials. Also, the CV and GCD curves of ZCO and G-ZCO in 2 M KOH and 2 M KOH + 0.1 M KFeCN are shown in Fig. S8–S10.†

The electrochemical performance of ZCO and G-ZCO was further evaluated *via* electrochemical impedance spectroscopy (EIS) acquired in the frequency range of 0.01 Hz to 10 000 Hz with an open circuit potential and an AC perturbation amplitude of 0.005 V to perceive additional information on the kinetics of ion diffusion and compare the electron migration taking place in neat KOH and KFeCN added electrolyte. The Nyquist plot displayed in Fig. 4e can be convoluted into four parts: (i) a pseudo-semicircle in the high-frequency region due to the resistance in the interfacial layer (*R*_IL_), and (ii) another semicircular arc in the mid-frequency region signifying charge transfer resistance (*R*_CT_). (iii) Warburg impedance (*W*) occurred at the mid-frequency region, which signifies the diffusional effect of electrolyte ions on the material's surface. (iv) Lastly, the almost vertical trend of the linear part of EIS in the low-frequency region deciphers the ideal capacitive nature.

Further Nyquist plots were fitted and then subjected to the Randles equivalent circuit model shown in Fig. S11.† ^34–36^ From Fig. 4e, we can simply observe the decrease in equivalent series resistance in the G-ZCO electrode when 0.1 M KFeCN was added to the 2 M KOH aqueous electrolyte. The inset displays a magnified image of the Nyquist plots, which also clearly indicates a minimal interfacial layer due to the smallest diameter of the semicircular arc for the G-ZCO electrode in the KFeCN added electrolyte.

The Bode plot depicted in Fig. 4f covers a similar frequency range, *i.e.*, from 0.01 Hz to 10 000 Hz. For an ideal capacitor, generally, the absolute phase angle is nearly equal to 90° at a very low frequency, while for faradaic pseudocapacitors and battery-type supercapacitors, the absolute phase angle deviates to <90° at a low-frequency area, which corresponds to the deviation to a pure capacitive behavior.^16^ In our graph, G-ZCO in 0.1 M KFeCN added to 2 M KOH possesses an impedance lower than that of the remainder of the systems in nearly all frequencies. The observed phase angle of G-ZCO at a lower frequency clearly indicates that all electrodes deviate from <90°, revealing their Faradaic-type charge storage mechanism.

The above clarified facts substantially prove that there is lesser resistance for G-ZCO in 2 M KOH + 0.1 M KFeCN, which favors the electrochemical kinetics. Apart from this, G-ZCO in 2 M KOH + 0.1 M KFeCN displayed a hump-like trait due to the excessive diffusion of the electrolyte ions on the surface of the electrode, which favors the rapid exchange of charges. Also, because of the overlapping of the charge transfer process (*i.e.*, capacitive and diffusive), completely weaker capacitive behavior was observed in the low-frequency region. The synergistic effect of dimensional tailoring and incorporation of redox mediators resulted in improved ionic conductivity, facile migration of electrons and ions, shortening of the diffusion path, and ultrafast chemical kinetics that substantially enhanced the supercapacitive activity.

A Randles–Ševčík (RS) plot was used to scrutinize the effect of scan rate on the voltammetric peak current, where linearity in the graph strongly indicates that the reaction occurring at the surface of the material is controlled by diffusion. The RS plot for G-ZCO is depicted in Fig. 4g, where a linear rise in the voltammetric peak current was observed with the scan rate, which confirms the reaction mechanism as being diffusion controlled. Low current density at a low scan rate was attributed to the formation of an impenetrable diffusion layer on the outer surface of the electrode material, which prevented the flux of the ions from the electrolyte solution to the material surface and resulted in higher current density.^37^ The RS plot also confirmed that the reaction mechanism is chemically reversible and is a diffusion-controlled redox process. Furthermore, similar characteristics were also observed for ZCO and G-ZCO in 2 M KOH electrolyte, which is shown in Fig. S12.†

Cycling stability is considered the most promising parameter for accessing supercapacitor performance in long-term potential application. Hence, the stability of G-ZCO was determined at a high current density of 10 A g^−1^. The obtained results in Fig. 4h show that 93% of the initial capacitance was retained through 6000 continuous repetitive cycles, and approximately 98% of coulombic efficiency was achieved. A slight rise in capacitance at 500–1000 cycles was noted, which may be due to the surface activation of the electrode in the alkaline solution. Also, the recorded before and after stability of the Bode and Nyquist plot in the inset of Fig. 4h provides evidence of the long-term stability of the material. These above-stated results provide strong evidence of the performance consistency of G-ZCO in redox mediator-included alkaline electrolyte, and indicate that it can act as an effective and practical path to amplify the supercapacitive activity of various active electrode materials by the synergistic mechanism of redox mediator addition and dimensional engineering.

### 3.5 Insight into the redox mediator mechanism

**Scheme 2:**
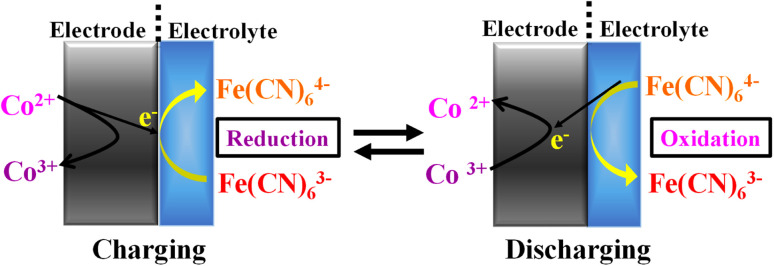
Mechanism of redox mediators at the electrode and electrolyte interface.

Although the performance of the G-ZCO was improved in the presence of KFeCN, the mechanism behind this remains unclear. Thus, in this section, we have clarified the mechanism that is taking place in the electrode and electrolyte interface by the incorporation of redox mediators into the aqueous electrolyte. Considering the charging state of G-ZCO, the redox-active element undergoes an oxidation reaction mechanism to form Co^3+^ from Co^2+^ by losing an electron, and the carriage of the lost electron governs the reaction rate. Furthermore, the supercapacitive performance of G-ZCO immensely improves once the elemental Co easily loses the electron. Adding potassium ferrocyanide (KFeCN) to the electrolyte solution quickly resolves the problem. The highly electrochemical reversible pairs of Fe(CN)_6_^3−^/Fe(CN)_6_^4−^ accept these ejected electrons *via* the reduction of KFeCN from 3^+^ to 4^+^. While charging, KFeCN 3^+^ ions act as an electron carrier and augment the supercapacitive activity. The entire process includes two series of reaction mechanisms represented in Scheme 2:Co^2+^–e^−^ → Co^3+^Fe(CN)_6_^3−^ + e^−^ → Fe(CN)_6_^4−^

Now considering the discharged state, the oxidation reaction has occurred from Fe(CN)_6_^4−^ to Fe(CN)_6_^3−^ by providing the required electron for the transition from Co^3+^ to Co^2+^, where Fe(CN)_6_^4−^ behaves as an electron donor to amplify the performance of the supercapacitor.^25,38–40^ Moreover, the potassium ferrocyanide ions play the vital role of electron shuttler in the charging–discharging process of G-ZCO by improving the electron relay in the reaction.

XRD experiments were also conducted to evaluate whether the KFeCN ion intercalates into the G-ZCO electrode. The graph in Fig. S13† demonstrates XRD after 1000 cycling tests of the G-ZCO electrode at 1 A g^−1^. No extra observable peaks for the KFeCN were found in the spectra. Also, two intense sharp peaks for Ni appeared, which were due to the usage of Ni foam as the current collector, and all the observable peaks for G-ZCO were retained. This experiment validates the lack of insertion of potassium ferrocyanide ion into G-ZCO, and the entire electron migration process occurred at the interface of the electrode and electrolyte.

### 3.6 Two-electrode electrochemical analysis

There has been recent research on flexible and wearable technologies. Electrode materials to be used in flexible supercapacitors should possess excellent specific capacitance and ultra-high-rate performance, along with impressive mechanical properties. The as-prepared dimensionally tailored heterostructure (G-ZCO) exhibited an impressive performance in ultra-flexible and high-performance supercapacitors. The FASD was constructed by utilizing G-ZCO as the positive electrode and A-C_60_ as the negative electrode, and PVA/KOH–KFeCN was used as the gel electrolyte cum separator for the facile migration of ions to prevent short-circuiting and leakage between the electrodes.

The physicochemical as well as electrochemical characterization of A-C_60_ in the aqueous electrolyte of 2 M KOH + 0.1 M KFeCN in a three-electrode setup has been elaboratively presented in the ESI text (Fig. S14 and S15†). To obtain the maximum probable and consistent potential window for the electrode materials, a charge and mass balancing method was adopted. For the final assembly of the FASD, the stored charges in the electrodes must be balanced by computing the active weight mass ratio, which was found to be 0.256, *via* the use of eqn (4).^9,41^4
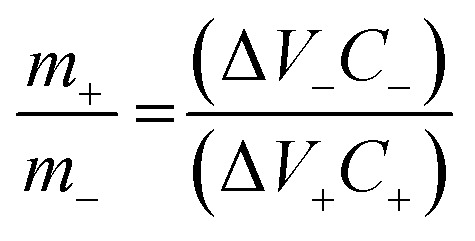


**Fig. 5:**
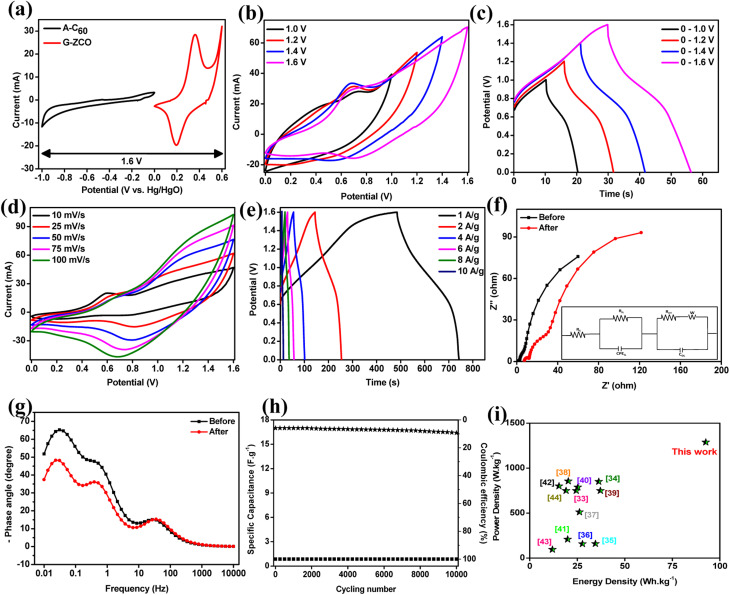
(a) Comparative CV plots of A-C_60_ and G-ZCO based on the three-electrode configuration at 10 mV s^−1^. (b) CV graphs of the flexible ASD at varied potential with a scan rate of 25 mV s^−1^. (c) GCD curves of the flexible ASD at varied potential with a current density of 6 A g^−1^. (d) CV plots of the flexible ASD at 1.6 V with diverse scan rates. (e) GCD curves of the flexible ASD at 1.6 V with varied current densities. (f) Fitted EIS curve of the flexible ASD before and after the cyclic stability test (the inset shows the Randles equivalent circuit diagram). (g) Bode plot of flexible ASD before and after the cyclic stability test. (h) Long-term cyclic stability and coulombic efficiency plot at an applied current density of 6 A g^−1^. (i) The energy density and power density of our work as compared with values from previous reports (comparative Ragone plot).

The active mass of *m*_+_ (G-ZCO) and *m*_−_ (A-C_60_) was calculated to be 1 mg and 3.906 mg, respectively, by computing the specific capacitance of both electrode materials (*C*_+_ and *C*_−_) *via* CV (Fig. 5a). The redox peak of G-ZCO and enclosed area of A-C_60_ show typical pseudocapacitive and EDLC-type behavior, respectively, by strongly suggesting that the FASD can provide a stable voltage window of 1.6 V. In the FASD, G-ZCO undergoes reversible faradaic reactions involving OH^−^ and K^+^ ions from the gel electrolyte, which were adsorbed/desorbed at the negative activated fullerene electrode while undergoing charging and discharging reactions. This reaction process was augmented due to the presence of KFeCN redox mediators in the gel electrolyte, and excellent rate capability and stability were exhibited.

To further evaluate the FASD's stability, CV and GCD curves were obtained at a varied potential window (*i.e.*, at 1.0, 1.2, 1.4, and 1.6 V). The CV curves recorded in Fig. 5b at a scan rate of 25 mV s^−1^ signify an increase in current response subjected to a more enclosed CV area with the increase in operating potential. In Fig. 5c, the series of GCD curves at varied potentials directly varies with the applied potential bias by displaying longer charging and discharging time graphs. Moreover, the reversible faradaic reaction and stable charge–discharge process were maintained at a high-voltage window of 1.6 V, indicating the satisfactory reversibility of the FASD. Fig. 5d shows CV curves recorded in a 1.6 V voltage window with a varied scan rate from 10 to 100 mV s^−1^. There are observable redox peaks at lower and higher sweep rates, which provide strong evidence of a reversible faradaic-type charge-storage mechanism from the simultaneous contributions of G-ZCO and A-C_60_.

Proof of the superb reversibility of the device was established by the unchanged CV curves at higher sweep rates. By fixing the potential bias at 1.6 V, a series of GCD tests at diverse current densities for the FASD was conducted. Fig. 5e shows a distinct plateau-like charging–discharging curve with no substantial *iR* drop. From the non-linearity traits of the discharge curves, there was validation for the faradaic redox reaction mostly occurring at the electrode–electrolyte interface, which is in agreement with the CV graphs. The coulombic efficiency at very low current densities (*i.e.*, 1 A g^−1^) was poor, but somehow increased at higher current densities. This type of trend can be attributed to spending less time at voltage boundaries, where irreversible reactions might hinder the performance and with slight deterioration of the electrolyte itself. There is satisfactory reversibility of the FASD in the entire applied potential range, and at a current density of 1 A g^−1^, it demonstrated the specific capacitance of 176 F g^−1^.

Apart from this, factors such as agglomeration of electroactive materials, formation of a solid electrolyte interface (SEI), occurrence of parasitic reactions such as partial water oxidation, slight self-discharging of the device due to the use of redox mediator, and increasing consumption of Fe(CN)_6_^3−^ during each discharging process can regulate the coulombic efficiency. A graph of the specific capacitance *vs.* current density for the FASD is shown in Fig. S16,† where a slightly low-rate performance is occurring at higher current densities. This is due to the slow ionic migration into the electrode materials where ions cannot access all the core and surface electroactive sites, resulting in low specific capacitance at relatively higher current densities.

From the EIS at the frequency response of 0.01–10 000 Hz, the Nyquist plot of the as-prepared ASD is displayed in Fig. 5f (the inset shows a fitted equivalent circuit). It can be convoluted into four sub-parts, a quasi-semicircle located in the high-frequency region due to the resistance in the interfacial layer (*R*_IL_), and another semicircular arc in the mid-frequency region, suggesting charge transfer resistance (*R*_CT_). Warburg impedance (*W*) also occurred at the mid-frequency region, which signifies the ion diffusional effect of electrolyte ions on the material's surface.^34–36^ An almost vertical trend of the linear part of the EIS in the low-frequency region evidently proves the ideal capacitive behavior of the FASD. The Bode plot displayed in Fig. 5g showing the deviations from the 90° phase angle demonstrates that the charge storage mechanism is dominated by the faradaic process.

The long-term cycling stability of the all-solid-state FASD was measured upon 10 000 cycles at a moderate density of 6 A g^−1^, and the results are displayed in Fig. 5h. The FASD retained 96.7% of its initial observed specific capacitance, which reveals the superior electrochemical reversibility and durability from the presence of redox mediators in the gel electrolyte and the two engineered nanostructured materials, which synergistically contribute to providing benchmark efficacy. This is adequately supported by the before and after EIS stability tests and Bode plots in Fig. 5f and g, respectively. Also, the use of A-C_60_ in negative electrodes can substantially contribute to device stability due to the enormous electron clouds of fullerenes and the high tensile strength from the sp^2^ hybridization.

Energy and power density are the major parameters for evaluating the efficiency of the electrode material, and thus, they are calculated by considering the GCD curves. The highest energy density value of 92.63 W h kg^−1^ was achieved at a power density of 1287.52 W kg^−1^, and even the highest power density of 1413 W kg^−1^ was achieved. Energy density *vs.* power density at each current density was calculated and is shown in Fig. S17.† Also, the Ragone plot in Fig. 5i shows that our current work outperforms some state-of-the-art studies by strongly demonstrating its utility in high-power and high-energy-related applications.^42–53^ Lastly, real-time energy storage was achieved by lighting a 3 mm LED light for a few seconds (Video SV1 in the ESI†).

**Fig. 6:**
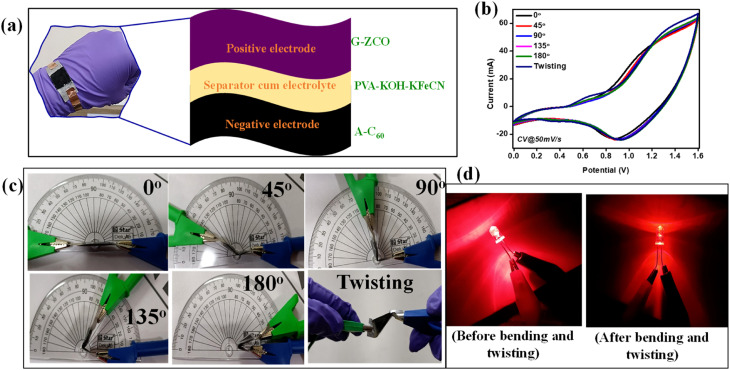
(a) Schematic illustration of all-solid-state flexible supercapacitor wearable device. (b) Obtained CV curves at a different angles of deformation and twisting. (c) Photographs showing the bending and twisting of the FASD. (d) Before and after comparison of illumination of an LED light subjected to 50 continuous bending and twisting tests.

The G-ZCO delivers incredible energy storage performance in 3- and 2-electrode measurements. Therefore, it is anticipated that it could perform well in terms of flexible supercapacitor application because of its dimensionally tailored porous materials architecture and redox-active gel electrolyte. To evaluate the mechanical strength and flexibility of the asymmetric supercapacitor device, the cell was folded into various angles, which strongly demonstrates its super-flexibility trait. Fig. 6a shows a schematic of a wearable device fixed on the wrist consisting of G-ZCO as the positive electrode and A-C_60_ as the negative electrode, with PVA-KOH-KFeCN as a gel electrolyte cum separator.

The CV and GCD curves shown in Fig. 6b and S18a† demonstrate that there is no obvious change in the curves after bending and twisting while being subjected to the high potential window of 1.6 V. Also, a graph of the specific capacitance *vs.* bending angles is shown in Fig. S18b,† where the specific capacitance of 17, 16.89, 16.85, 16.82, 16.77, and 16.75 F g^−1^ at 6 A g^−1^ was obtained at bending of 0°, 45°, 90°, 135°, 180° and twisting states, respectively. Fig. 6c and d show photographs of the bending and twisting of the FASD and before and after comparison of the illumination of an LED light subjected to continuous 50 bending and twisting tests, respectively, where the illumination continues after numerous bending and twisting cycles. Fig. S18c–h† shows digital photographs of the light illumination from the 3 mm red LED light that occurred by connecting two asymmetric devices in series, with their respective pictorial bending representation. No deviation in the luminescence of the LED was noted at various mechanical deformation angles, which strongly indicates its performance consistency under extreme mechanical environments.^54^

A comparison of various ZCO- and GaN-based electrodes with our work is presented in Table S1,† which demonstrates that our current work outperforms the presented state-of-the-art literature. The fabricated ultra-flexible and wearable all-solid-state asymmetric supercapacitor device is extremely stable at high operating voltage as well as during rigorous deformation and twisting. Thus, it can be diversely applied in flexible and wearable electronics and used as a model for designing miniature next-generation hybrid supercapacitors.

### 3.7 Post-performance characterization

To validate the stability of our material, crystal phase structure, chemical properties, and morphology were evaluated after 6000 continuous charge–discharge cycles. The XRD and FTIR spectra depicted in Fig. S19† distinctly clarify that no significant change occurred even after 6000 continuous charge–discharge cycles compared to those of the freshly prepared samples. No extra peaks or stretching contributions from the metal oxides and hydroxides were observed. Furthermore, FE-SEM strongly revealed no notable change in the morphology or structure of the as-formed particles. The distinct pores and 1D/2D heterostructures without any damaged pores or cracks are also clearly visualized in Fig. S20a and b.† The EDX (Fig. S20c†) also confirmed that the proper elemental percentage was retained after the stability test without compromising the performance of the materials.

## 4. Conclusion

We augmented the supercapacitive performance of a G-ZCO nanocomposite by utilizing the synergistic effect of dimensional engineering and incorporation of a potassium ferrocyanide redox mediator. Redox reaction and electron relay between Fe(CN)_6_^3−^/Fe(CN)_6_^4−^ immensely accelerated the transition between Co^2+^ and Co^3+^ by improving the electro-kinetics at the electrode and electrolyte interface, and thus realizing improved electrochemical properties. Compared with other reported ZCO-based supercapacitors, the as-developed FASD displayed enhanced capacitance and excellent stability as well as a higher energy and power density. Because the performance of the FASD was the best reported to date, this confirmed the significant potential of this proposed technique for use in efficient energy storage devices. Our results demonstrate an appealing and effective strategy to merge dimensionally tailored pseudocapacitive materials with redox electrolytes to enhance the energy storage performance, thus paving the way toward next-generation high-performing supercapacitor devices.

## Author contributions

Ritik Mohanty: conceptualization, methodology, investigation, visualization, writing-original draft preparation and writing-editing, Kaushik Parida: writing-reviewing and editing, supervision, writing-reviewing and editing, supervision.

## Conflicts of interest

There are no conflicts to declare.

## Supplementary Material

NA-005-D3NA00279A-s001

NA-005-D3NA00279A-s002
