# Supplementary material for: Redox mediator-enhanced charge storage in dimensionally tailored nanostructures towards flexible hybrid solid-state supercapacitors
Source: Nanoscale Adv. 2023 Aug 9;5(17):4521–35. doi: 10.1039/d3na00279a (PMC10448346; doi:10.1039/d3na00279a)
Supplement: NA-005-D3NA00279A-s002 [file NA-005-D3NA00279A-s002.pdf]

Electronic Supplementary Material (ESI) for Nanoscale Advances.
This journal is © The Royal Society of Chemistry 2023

Redox mediator enhanced charge storage in dimensionally tailored nanostructure towards flexible hybrid solid-state supercapacitors

Ritik Mohanty[a], Kaushik Parida[b*], Kulamani Parida[a*]

[a]Centre for Nanoscience and Nanotechnology, Siksha 'O'Anusandhan (Deemed to be University), Bhubaneswar-751030, Odisha, India

[b]Department of Polymer & Process Engineering, Indian Institute of Technology Roorkee, Saharanpur Campus, Uttarakhand-247001, India

***Corresponding author**

E-mail: Kulamani Parida (kulamaniparida@soa.ac.in) & Kaushik Parida (kaushik.parida@pe.iitr.ac.in)

Supporting Information:

Materials Characterization:

Phase and structure of the materials were evaluated by Powder X-ray Diffraction technique (Cu Kα radiation source, λ = 0.154 nm) in the scanning window 2θ = 20−80° having 40 kV voltage and 40 mA current of X-ray source [*Rigaku-Ultima IV*]. Fourier transform infrared (FTIR) spectrum was taken by taking KBr pellet as reference [*JASCO FT/IR-4600*]. A confocal Raman spectrometer [*RENISHAW in Via*] was used for the Raman analysis (520 nm laser irradiation by the solid-state laser). The outer morphology was investigated by FE-SEM (Field emission scanning electron microscope equipped with EDX) having an acceleration voltage of 20 kV by *ZEISS Gemini SEM 300*. The outer morphology of the C_{60} and A-C_{60} is measured by *MIRA3 TESCAN*. The inner morphology was analysed by *FEI Tecnai G2 F30 TEM* (Transmission electron microscope) having an acceleration voltage of 300 kV. The elemental compositions of the investigated composites were analysed through XPS (*Thermo Fischer Scientific ESCALAB Xi+*) using Al Kα radiation of 200 W and all the XPS curve fitting was accomplished by using CASA XPS software. All the electrochemical characterizations were done by the *IVIUM n STAT* single-channel electrochemical analyser. The surface area analysis was conducted in Quantachrome® ASiQwin by degassing the samples at 200 ^{0}C for 8 h.

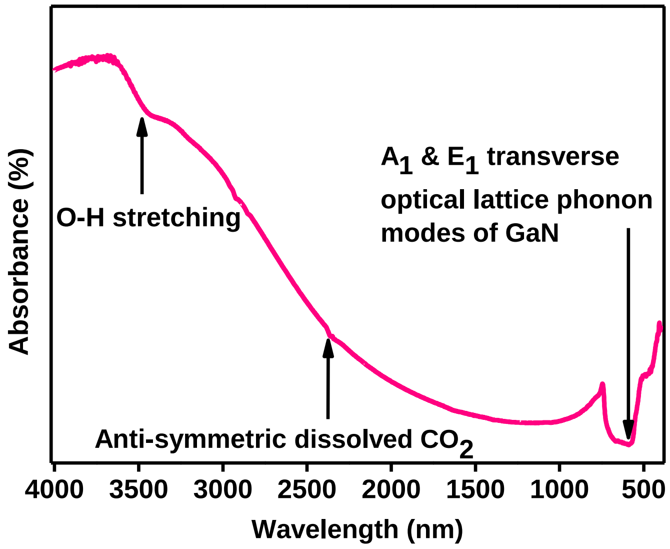

Fig. S1 FTIR spectrum of GaN.

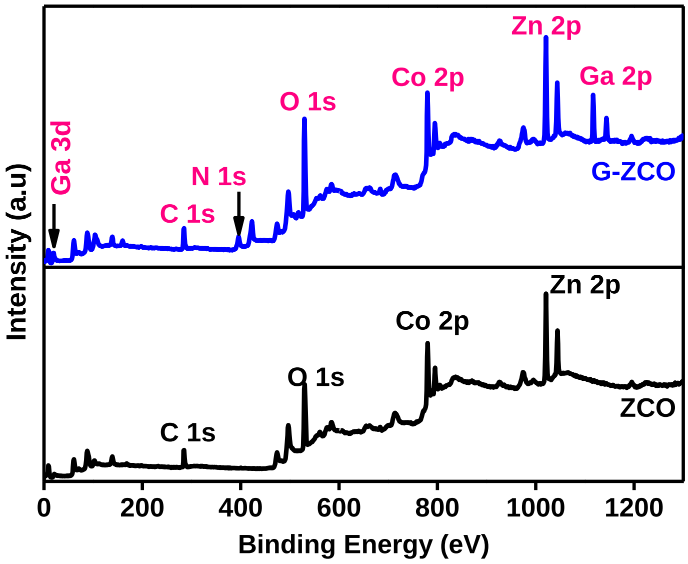

Fig. S2 XPS survey spectra of ZCO and G-ZCO.

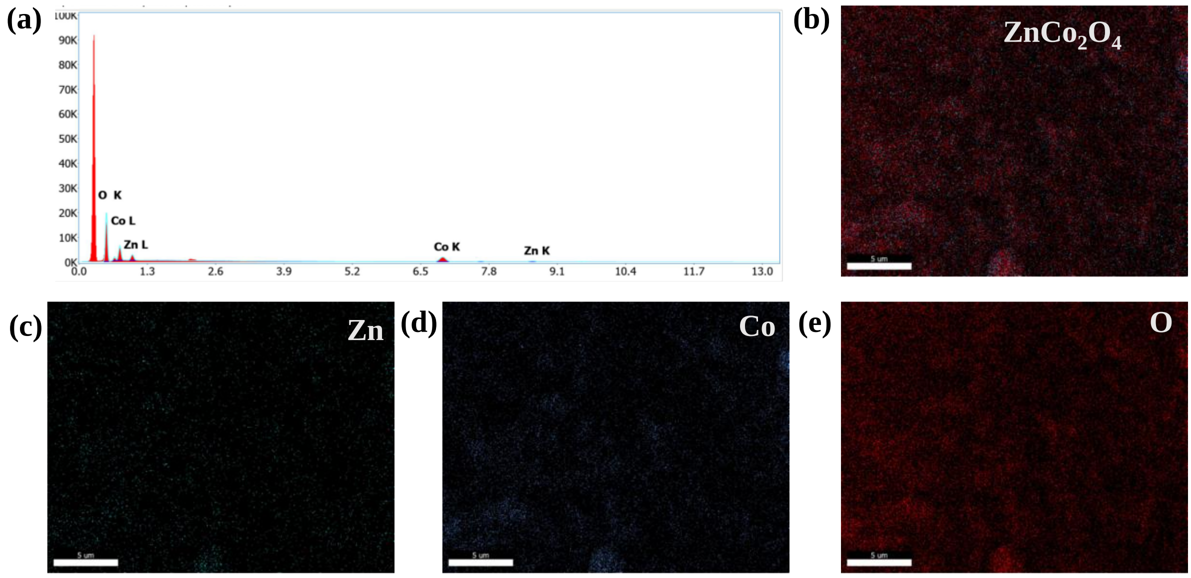

Fig. S3 (a) EDX spectrum and (b) corresponding colour elemental mapping for ZCO.

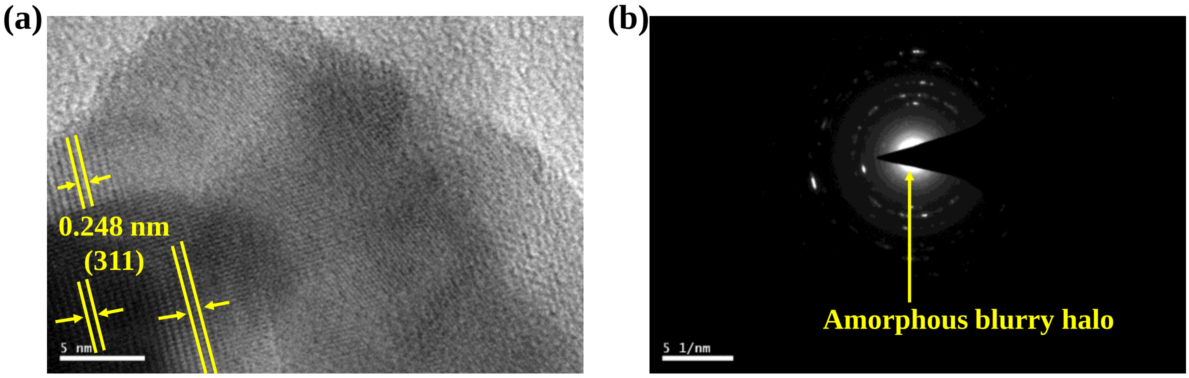

Fig. S4 (a) HRTEM image and (b) SAED pattern of ZCO.

Electrochemical characterization:

All the electrochemical analysis (three-electrode) are performed by the IVIUM n STAT multichannel electrochemical analyser by taking drop casted samples on the Ni foam as working electrode, Pt wire as counter, and Hg/ HgO as reference electrode and 2 M KOH and 2 M KOH + 0.1 M KFeCN as aqueous electrolyte. Before material casting, Ni foam was washed thoroughly with 0.1 M HCL solution, DI water, and acetone for 20 min each under continuous ultrasonication (frequency of 40 kHz) to get rid of the formed oxide layer. Subsequently, the mixture of working electrode material, carbon black (as a conductive agent), and Nafion (as a binder) in a weight ratio of 75:15:5 was dispersed in an adequate amount of a pure anhydrous ethanol solution and the slurry was drop-casted over a working area of 1 × 1 cm^2 and vacuum dried overnight at 80 °C. Lastly, 10 MPa hydraulic pressure of was applied for a few secs to form a thin film of active material coated Ni foam working electrodes. To construct a flexible asymmetric cell, G-ZCO was taken as positive and activated fullerene (A-C_{60}) as the negative electrode material, and Ni foam as a current collector. The device was constructed by assembling those positive and negative electrodes using a PVA-KOH-KFeCN gel electrolyte as both electrolyte and separator. Here the Ni Foam facilitates the formation of a flexible supercapacitor with excellent stability under deformations.

Electrochemical performance estimation for both two and three electrode system:

The specific capacitance of the as-synthesized electrode materials was evaluated from the galvanostatic charge-discharge (GCD) curves by considering the below-mentioned equation. [1,2]

$$C_{sp} = \frac{2}{mV^2}\int iV(t)dt \quad \cdots\cdots\cdots\cdots\cdots\cdots\cdots\cdots (1)$$

where *iV(t)dt* is the enclosed area under the discharge curve, *m* is the mass loading of the active electrode material and *V* is the working voltage window.

Further, the Specific capacitance can be calculated from cyclic voltammetry (CV) graphs by the given formula

$$C_{sp} = \frac{\int I \, dv}{[mk(\Delta V)]} \, F.g^{-1} \cdots\cdots\cdots\cdots\cdots\cdots\cdots\cdots (2)$$

here *I* is the enclosed area under the CV curve, *k* is the sweep rate and ΔV is the working voltage window.

The energy density (E_D) and power density (P_D) were evaluated from the following equation considering GCD curves of fabricated flexible all-solid-state asymmetric supercapacitor device

$$E_D = \frac{1}{m}\int iV(t)dt \cdots\cdots\cdots\cdots\cdots\cdots\cdots\cdots (3)$$

$$P_D = \frac{E}{t} \cdots\cdots\cdots\cdots\cdots\cdots\cdots\cdots (4)$$

where *m* is the mass of the total active material in the device (i.e., the sum of the individual electrode masses in both the positive electrode and negative electrode) and *t* is discharging time.

Coulombic efficiency (η) was also evaluated by using the following equation by taking the ratio of discharging (t_d) and charging (t_c) time while there is no significant change in charging current densities.

$$\eta = \left(\frac{t_d}{t_c}\right) * 100 \cdots\cdots\cdots\cdots\cdots\cdots\cdots\cdots (5)$$

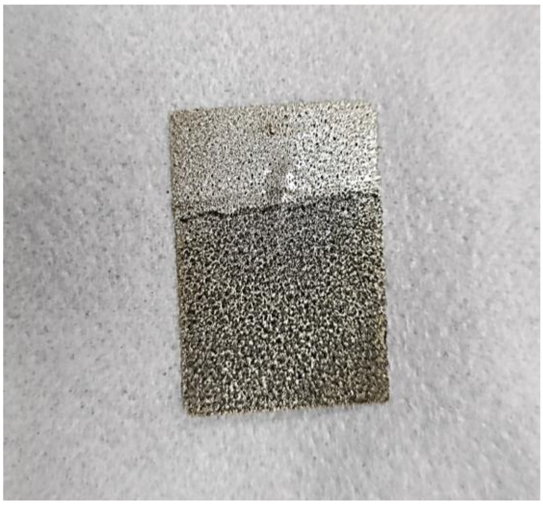

Fig. S5 Digital image of Ni foam as a current collector and drop casted G-ZCO sample as active electrode material.

Effect of various KFeCN amounts in the super capacitive performance of G-ZCO:

In our study, we have focused on investigating the performance of the supercapacitor in the electrolyte composition of 2 M KOH + 0.1 M KFeCN. The selection of this particular electrolyte composition was based on its favourable electrochemical properties and the balance it offered between specific capacitance, and long-term stability. We chose the 0.1 M KFeCN concentration based on the availability of literature reports that support its suitability for supercapacitor applications. Numerous studies have reported excellent performance in this concentration range, establishing it as a standard reference for benchmarking the performance of supercapacitors. By utilizing this concentration, we aimed to facilitate direct performance comparisons with other reported results, enhancing the reproducibility and comparability of our findings. As per your suggestion, we understand the importance of investigating the performance of electrolytes with different KFeCN concentrations. Varying the KFeCN concentration can have an impact on several aspects of supercapacitor performance, including specific capacitance, and stability. We acknowledge that studying the performance of our supercapacitor in electrolytes with different KFeCN concentrations would provide valuable insights into the optimal composition for achieving desired performance characteristics. Thus, we have currently varied the KFeCN concentrations, such as 0.05 M, 0.1 M, 0.2 M, 0.5 M, 1 M, and 2 M and conducted electrochemical measurements to systematically investigate the effect of varying these experimental results provide strong metrics to evaluate the performance across a range of concentrations and provide a comprehensive understanding of the KFeCN concentration's impact on the super capacitive behaviour of G-ZCO. For comparison purpose,

we have also given the CV curves (Fig. S6a) and explained in the modified manuscript to gain insight into how the concentration of KFeCN affect performance in terms of specific capacitance and stability. With gradual percentage increment of KFeCN in the KOH displays an appreciable increase in the area of cyclic voltammogram along with a higher current value. Compared to one with any redox mediator, the presence of KFeCN of any concentration beyond 0.5 M redox additive gives a larger enclosed area. Further, increasing the redox additive amount to 2 M no noticeable change is observed in the enclosed area of the CV curve, however, much more capacitance loss was observed after 1000 cycles (Fig. S6b) as well as poor rate capability. Hence 0.1 M KFeCN is chosen as the best and optimum concentration for further electrochemical analysis and measurements by considering its balanced trait such as offering high specific capacitance as well as stability.

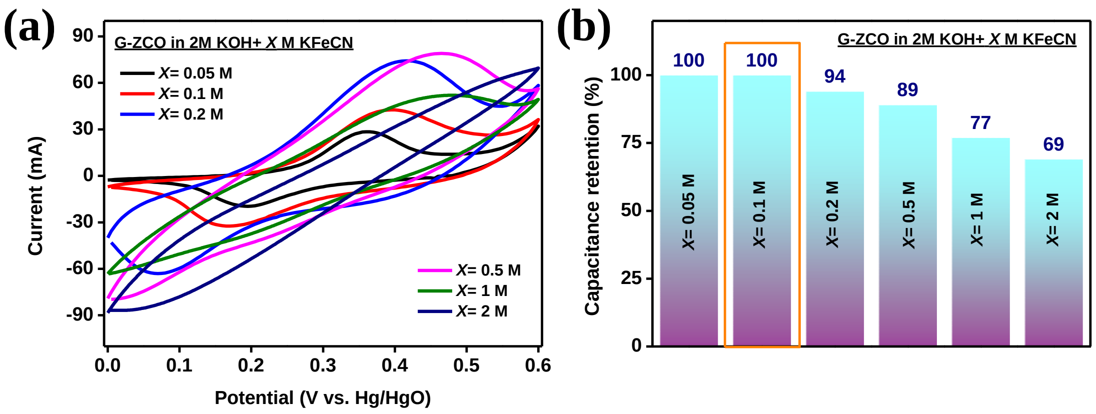

Fig. S6 (a) CV of G-ZCO in different KFeCN added electrolytes in three-electrode configuration, (b) capacitance retention of G-ZCO in different KFeCN added electrolytes after 1000 CV cycles at 10 mV.s^{-1}.

When the KFeCN concentration is lower (i.e., 0.05 M) the contributions from the redox reactions from KFeCN is lower resulting in a low specific capacitance. Further, the performance of the electrode material tends to decrease when the KFeCN amount is greater than 0.1 M, which indicates that an extra amount of KFeCN is also not favorable for the general improvement of capacitive nature at a high rate. The reason for this is concentration polarization and pore-blocking effect when KFeCN content is high, which leads to slow chemical redox reaction kinetics. So, from the above, KFeCN concentration has a significant influence on the specific capacitance, coulombic efficiency, and rate property. The low rate capability can be attributed to the fact that at higher specific currents the obtained time constraints limit the diffusion of $[Fe(CN)_6]^{3-}$ ions through the electrode and electrolyte

interface. While at lower specific currents $[Fe(CN)_6]^{3-}$ ions would have adequate time for accessing the interface, making it capable of delivering a higher specific capacitance value. Higher KFeCN concentrations facilitate high specific capacitance, and coulombic efficiency, but worsen the rate property and electrochemical stability. Now coming to the fact that how the concentration of KFeCN affect performance, in supercapacitors, KFeCN is often used as a redox-active component in electrolyte to facilitate the charge storage and release processes. This can modify the electrochemical behavior of the supercapacitor by considering below-mentioned points such as pseudocapacitive contribution, the occurrence of redox reaction, stability, electrode, and electrolyte kinetics.

a) <u>Pseudocapacitive contribution:</u> KFeCN can contribute to the overall pseudocapacitance of the supercapacitor. Pseudocapacitance arises from fast and reversible faradaic redox reactions occurring at the electrode-electrolyte interface. As the concentration of KFeCN increases, the pseudocapacitive contribution tends to increase, leading to higher overall capacitance and potentially enhanced energy storage capabilities.

b) <u>Occurrence of redox reaction:</u> KFeCN undergoes redox reactions, which can influence the performance of the supercapacitors. The redox processes involving KFeCN can provide additional redox pairs which can take part in augmenting the charge storage mechanisms during Faradic reactions.

c) <u>Stability:</u> Somehow the concentration of KFeCN can influence the stability of the electrolyte and the electrode materials. Excessive concentrations of KFeCN may announce additional side reactions or improve the chances of undesired chemical reactions, which can greatly impact the long-term stability and cycling performance of the supercapacitor. Finding the proper balance of KFeCN concentration is crucial to maintain stability while maximizing electrochemical performance.

d) <u>Electrode and electrolyte kinetics:</u> The concentration of KFeCN regulates the kinetics of the redox reactions occurring at the surface of the electrode with the electrolyte ions. Optimal concentrations of KFeCN can provide a greater number of redox-active centres, potentially leading to improved charge transfer rates and improved electrochemical performance. However, extremely high concentrations may lead to improved internal resistance or diffusion limitations, which affects the performance negatively.

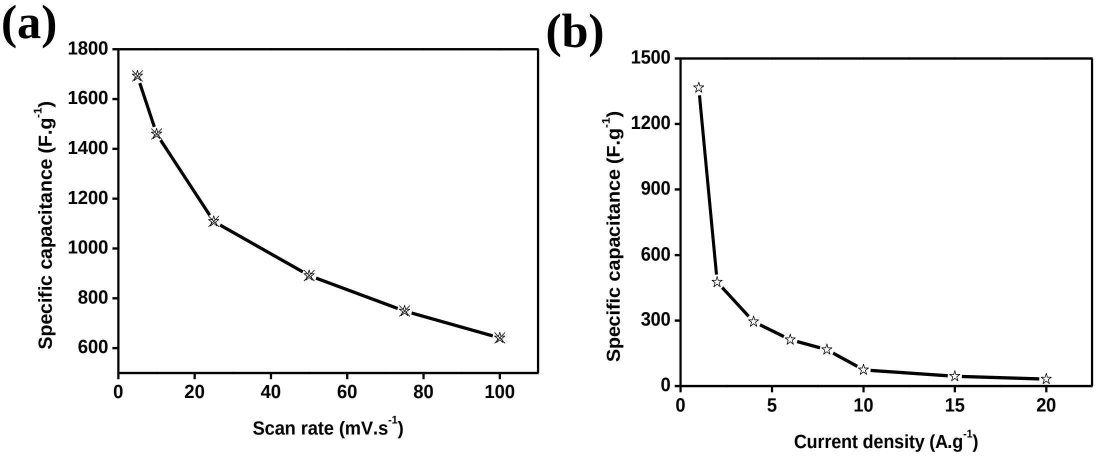

Fig. S7 (a) calculated specific capacitance at different scan rates, (b) calculated specific capacitance at varied current densities.

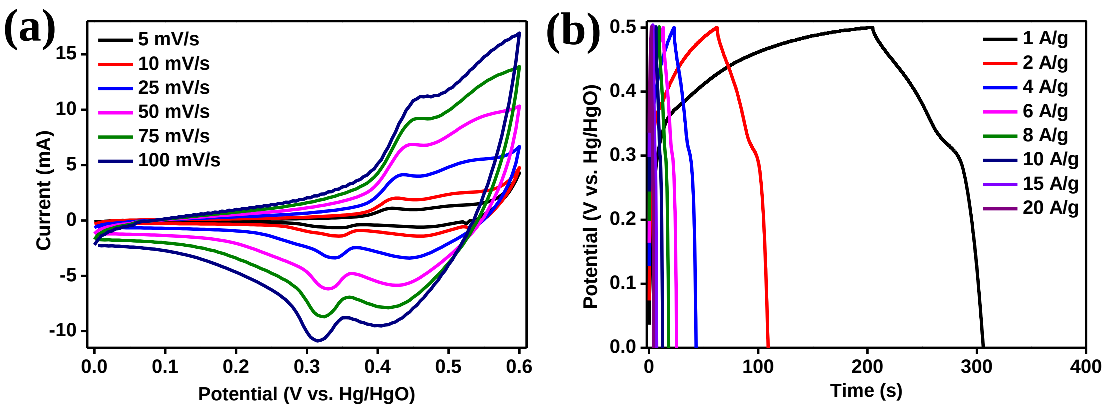

Fig. S8 (a) CV and (b) GCD curves of ZCO in 2 M KOH.

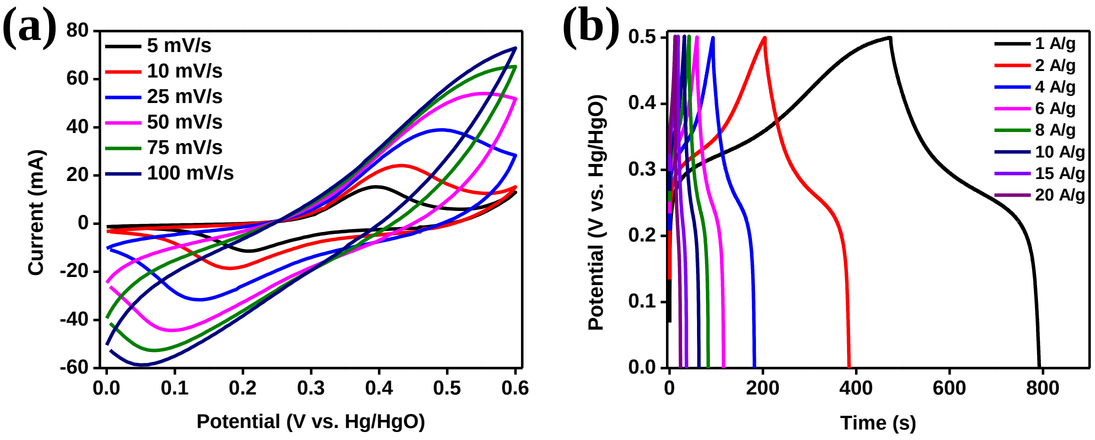

Fig. S9 (a) CV and (b) GCD curves of G-ZCO in 2 M KOH.

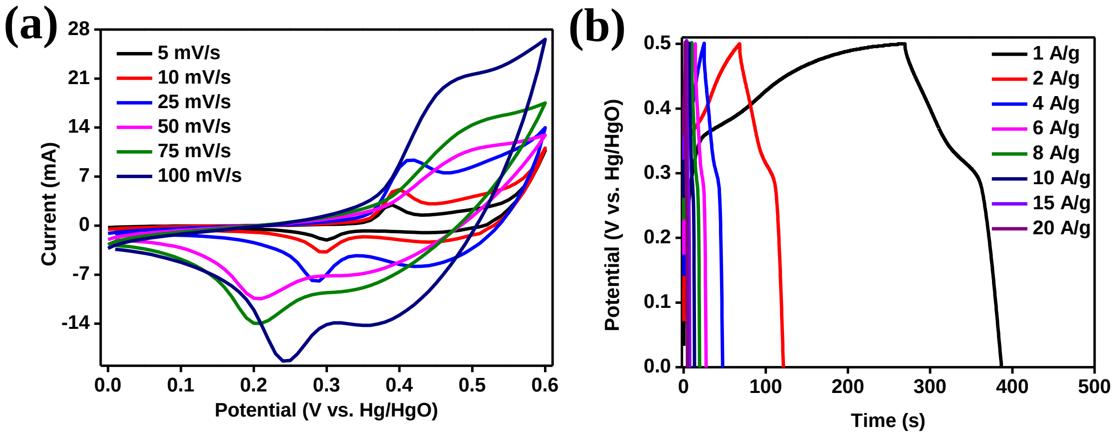

Fig. S10 (a) CV and (b) GCD curves of ZCO in 2 M KOH+ 0.1 M KFeCN.

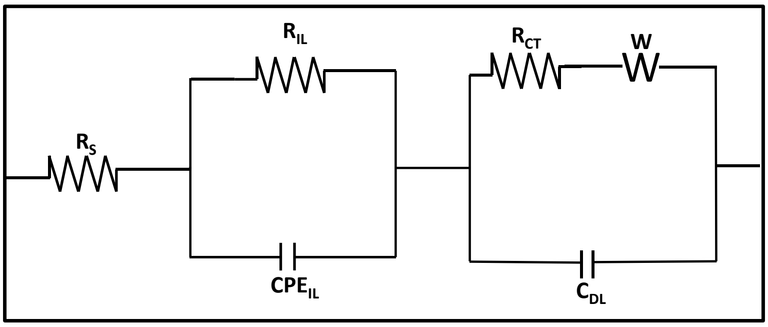

Fig. S11 Randles equivalent circuit for 3 electrode EIS measurements (Nyquist plot).

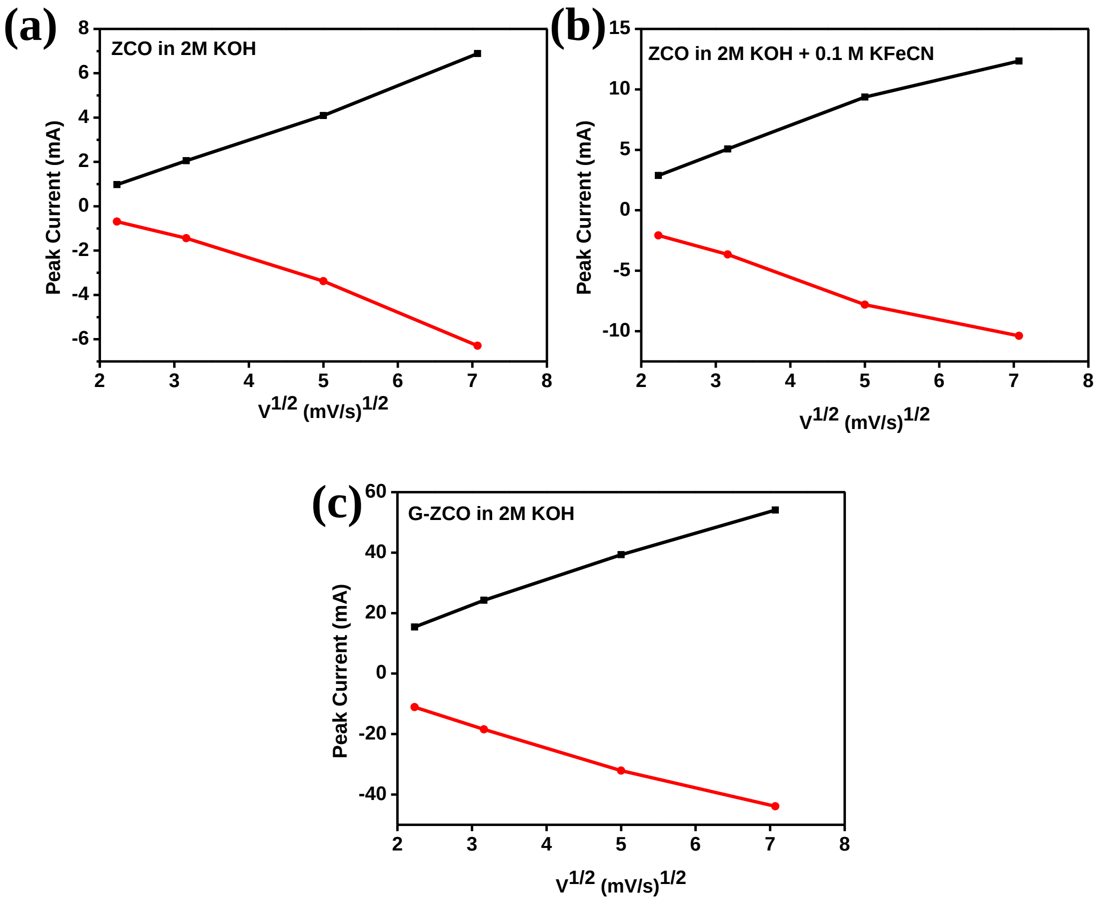

Fig. S12 (a-c) RS Plot of ZCO and G-ZCO.

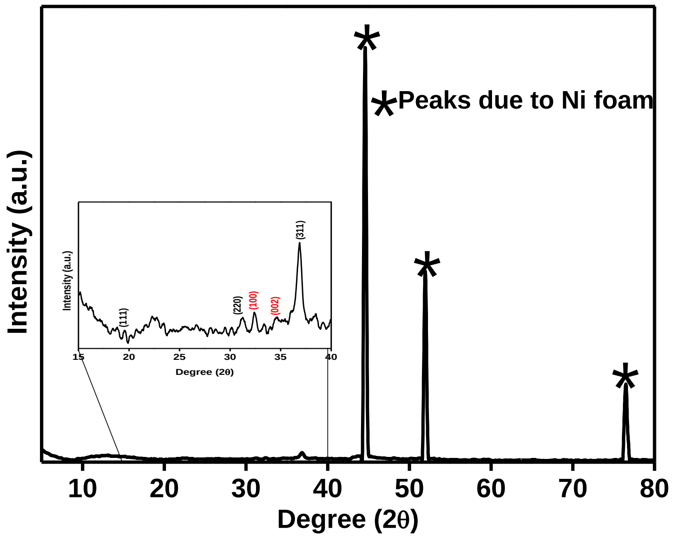

Fig. S13 XRD Plot of G-ZCO electrode after 1000 GCD cycles in 2 M KOH + 0.1 M KFeCN electrolyte.

Physicochemical and electrochemical properties of negative electrode material (A-C_{60}):

A super flexible and wearable all-solid-state asymmetric hybrid supercapacitor device (FASD) is fabricated by utilizing G-ZCO as the positive electrode and activated fullerene (A-C_{60}) as the negative electrode. Here A-C_{60} is chosen as the negative electrode material in the hybrid supercapacitor owing to its impressive mechanical and electrochemical properties along with superb stability in extreme mechanical deformations. So, in this section, we have briefly provided the physicochemical as well as electrochemical characterization of A-C_{60} in the aqueous electrolyte of 2M KOH + 0.1M KFeCN in a three-electrode setup. In this work, A-C_{60} is fabricated by the thermal functionalization of the C_{60} powder taking KOH as the activator. The XRD peaks represented in Fig. S14a shows the diffraction peaks for both the activated as well as pristine sample. A-C_{60} shows diffraction peaks at 10.85°, 17.83°, 20.83°, 21.72°, and 28.28°, indexed to crystal planes of (111), (220), (311), (222), (420) which is well agreement with the cubic fullerene having space group F (JCPDS No. 44–0558)[3]. Owing to surface functionalization as well as partial graphitization peak broadening is noticed as compared to neat C_{60} which strongly suggests its semi-crystallinity nature.

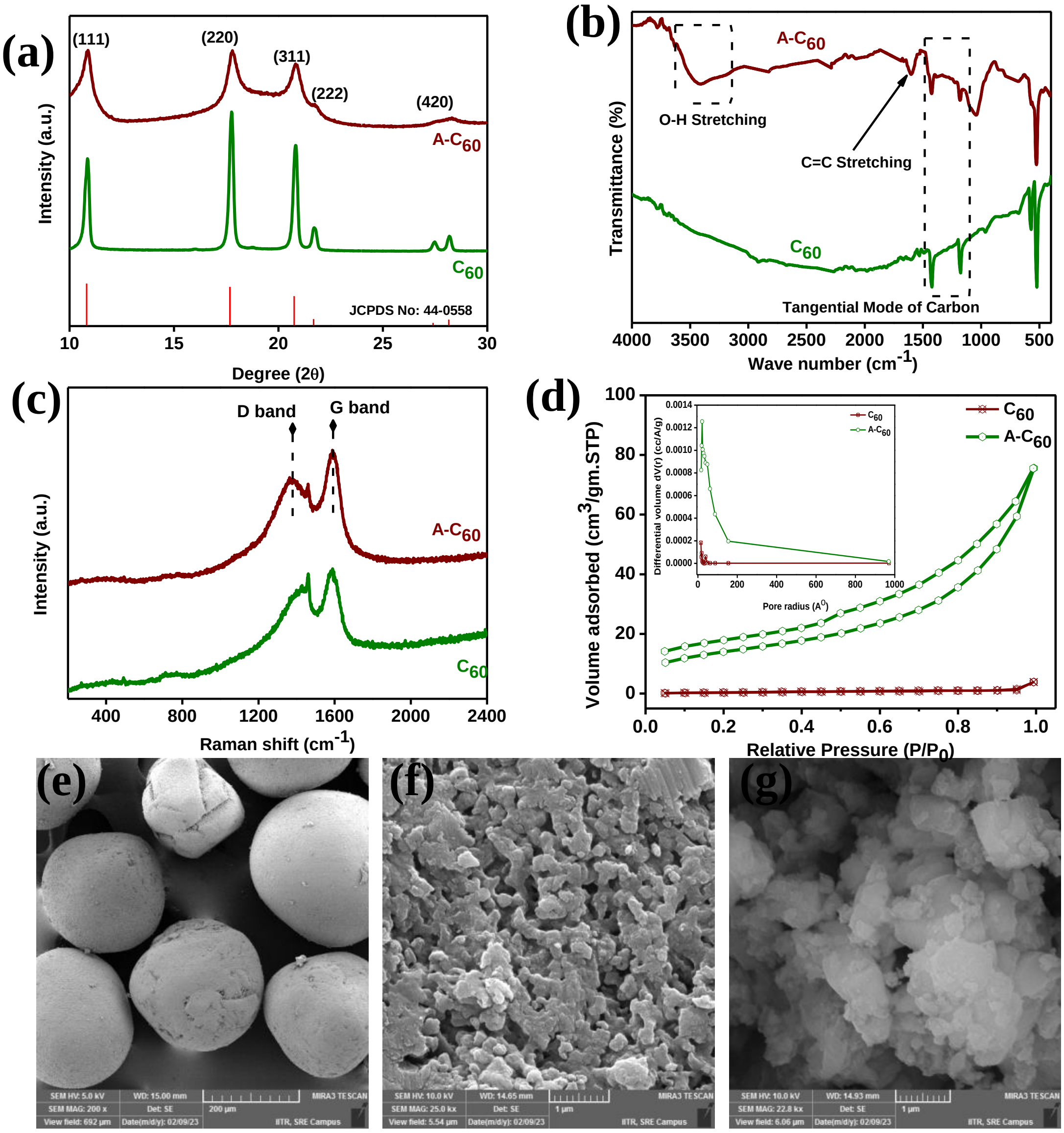

Fig. S14 (a)XRD, (b) FTIR, (c) Raman, (d) Isotherm plots (insert showing corresponding pore size distribution) for C_{60} and A-C_{60}, (e) SEM image of C_{60}, (f) magnified SEM image of C_{60} and (g) magnified SEM image of A-C_{60} showing porous spherical structure.

FTIR analysis of C_{60} and A-C_{60} shown in Fig. S14b clarified the phase identity and presence of functional groups. In both the samples active vibration modes of F_{1u} symmetry are observed at ~524, ~573, ~1179 and ~1427 cm^{-1} due to the radial displacement of carbon atoms for the two bands of lowest wavenumber and tangential modes of carbon atoms for the two modes above 1000 cm^{-1}.[3] Likewise, post thermal functionalization A-C_{60} shows two extra vibrational modes at ~1603 and ~3414 cm^{-1} subjected to C=C (or attributed to water bending) and O-H stretching respectively which demonstrates about the increase in hydrophilicity of the

material.[3] Further Raman analysis showed the two active vibrational bands as the D band and G band at ~1383 and ~1591 cm^{-1} (Fig. S14c). The evaluation-specific surface area and pore features are examined by N_2 sorption isotherms. The P/P_0 hysteresis curve of A-C_{60} is greater and wider than sole C_{60} revealing smaller mesopores as well as a high specific surface area of thermally activated sample. The surface area of A-C_{60} is found to be 51 $m^2.g^{-1}$ which will provide a large number of electroactive pores and surfaces for enhanced energy storage ability (Fig. S14d). Furthermore, the fine mesoporous structure of A-C_{60} has a pore radius of 19.08 Å which can accumulate a huge amount of electrolyte ions inside its pores to accomplish a quick diffusion reaction to interact with the core of the electrode surface. Furthermore, scanning electron microscopy (SEM) techniques were used to investigate the external morphology of the material where the C_{60} possesses a distinct spherical nature (Fig. S14e). Also, the high-resolution micrographs represented in Fig. S14f and g show post-thermal functionalization of C_{60} (i.e., in A-C_{60}) irregular mesopores, and the roughened surface is observed rather than pristine C_{60}. Substantially it can increase the specific surface area value; thus, resulting in improved electrochemical kinetics, high power density, and specific capacitance.

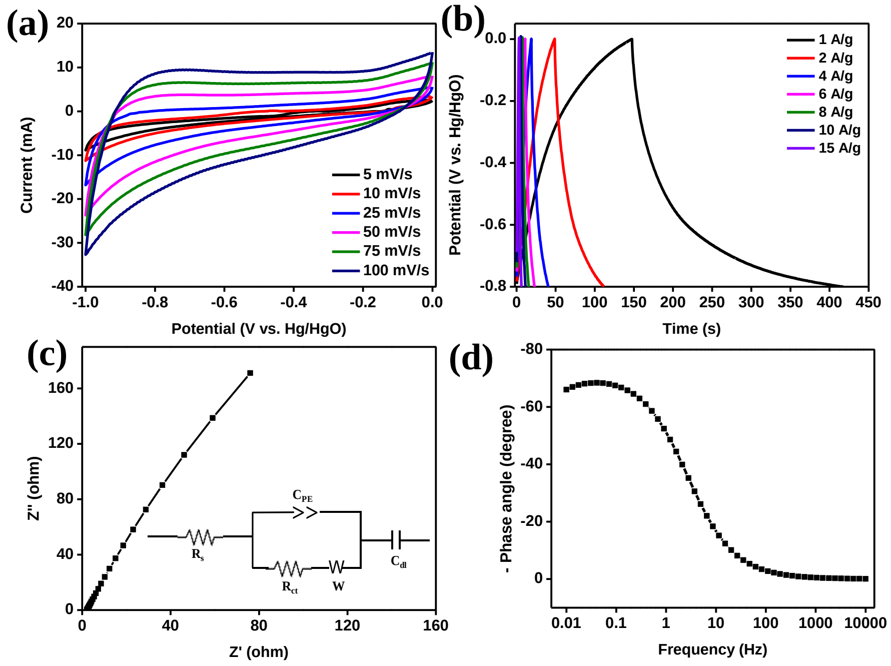

Fig. S15 Electrochemical characterization of A-C_{60} in 2 M KOH + 0.1 M KFeCN electrolyte in three-electrode setup representing (a) CV curves at diverse sweep rates, (b) GCD curves recorded at different applied current density, (c) Nyquist plot and (d) Bode plot in the frequency range of 0.01 Hz to 10000 Hz.

The electrochemical properties of A-C_{60} electrodes were analyzed in the aqueous electrolyte of 2 M KOH + 0.1 M KFeCN in a three-electrode setup. CV curves represented in Fig. S15a are recorded in the potential range of 0 to -1.0 which shows the typical quasi-rectangular shape. This signature quasi-rectangular nature of the CV and quasi-triangular GCD curves (Fig. S15b) clarifies the charge storage mechanism of the A-C_{60} is dominated by electric double-layer formation having a non-Faradic charge storage mechanism. The highest capacitance of 435 $F.g^{-1}$ is obtained at 5 $mV.s^{-1}$ in the aqueous mixed electrolytes due to the high surface area, conductivity, short diffusion length, and stable nature of the active material. Further, the EIS and Bode plot represented in Fig. S15c and d shows a nearly ideal capacitive nature. The Nyquist plot of A-C_{60} shows a linear straight line having a steady slope proportional to the real axis and a tiny ill-defined arc at high frequency, which strongly proves the ideal capacitive nature of the materials. Furthermore, a slight deviation in the behavior of the pure capacitor is noticed at the low-frequency range of the Bode plot. Lastly from the physicochemical and electrochemical characterization, it is well verified that the A-C_{60} can act as an efficient negative electrode material to provide augmented electrochemical performance and stability to the all-solid-state hybrid supercapacitor device.

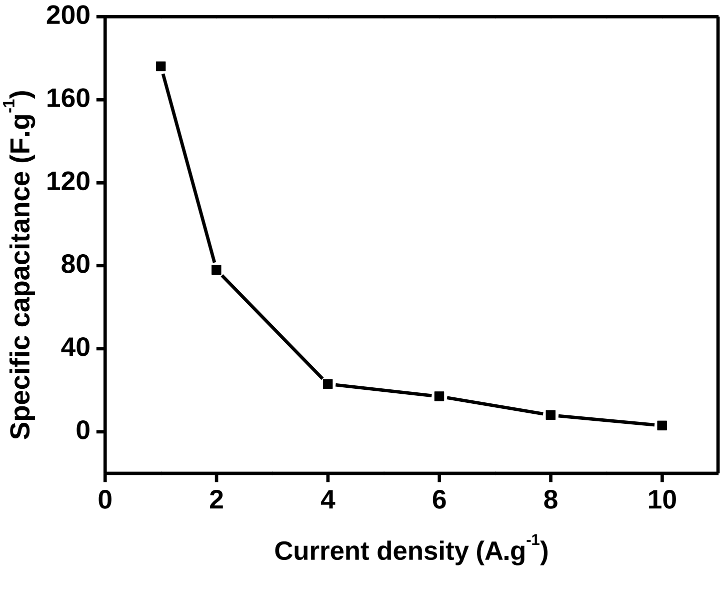

Fig. S16 Current density vs. specific capacitance curve for the FASD.

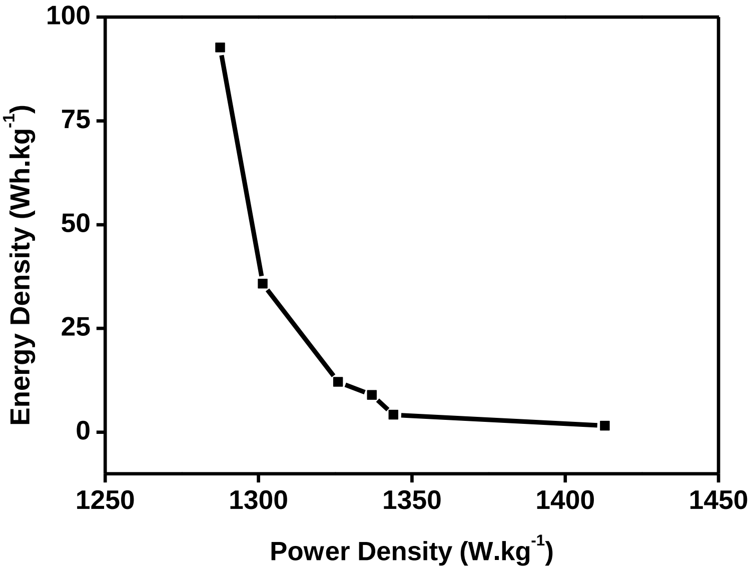

Fig. S17 Ragone plot of our work.

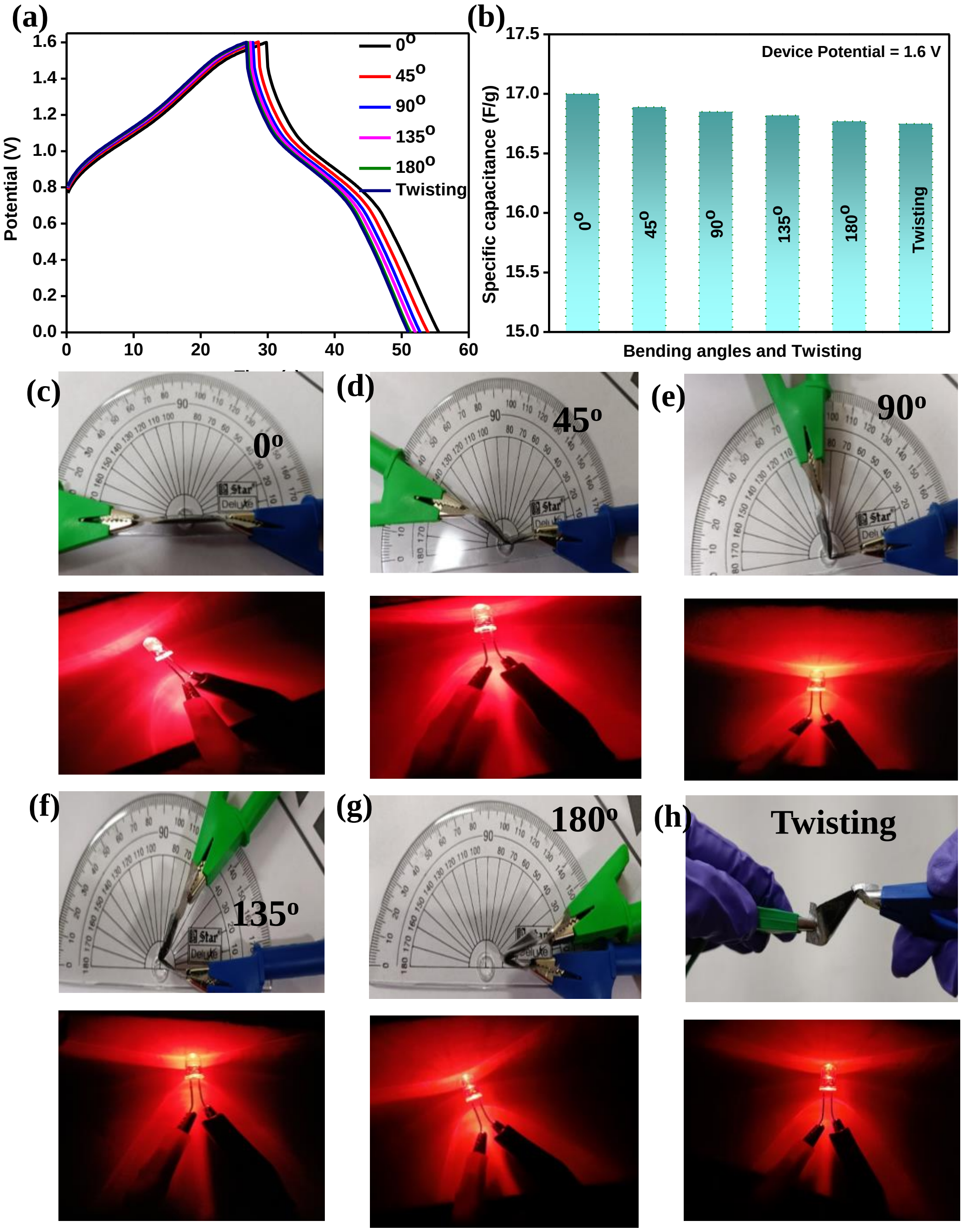

Fig. S18 (a) GCD cycles at 6 $A.g^{-1}$ subjected to various bending and twisting angles, (b) Bending angles vs. specific capacitance obtained at 6 $A.g^{-1}$ of the as-prepared device. and (c-h) illuminating a red LED using two fabricated devices connected in series subjected to respective bent and twisted angles as displayed.

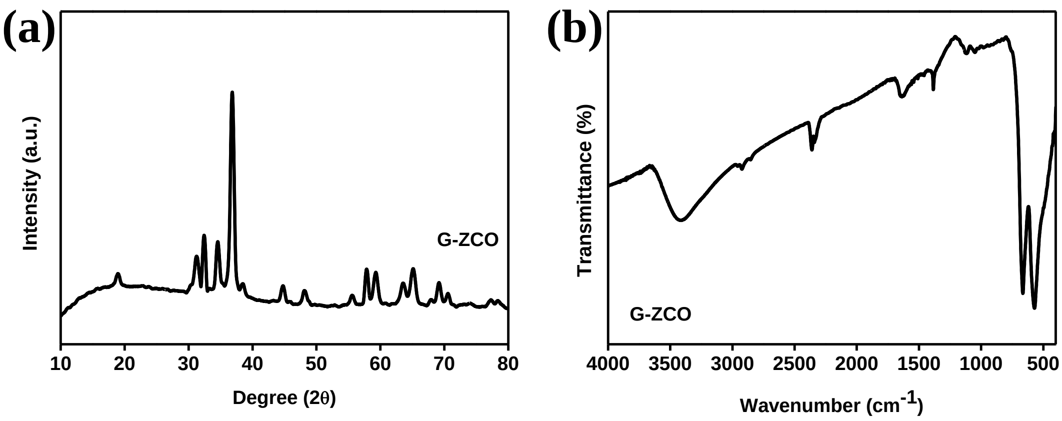

Fig. S19 (a) XRD and FTIR spectrum of G-ZCO for re-usability test after 6000 continuous charge-discharge cycles.

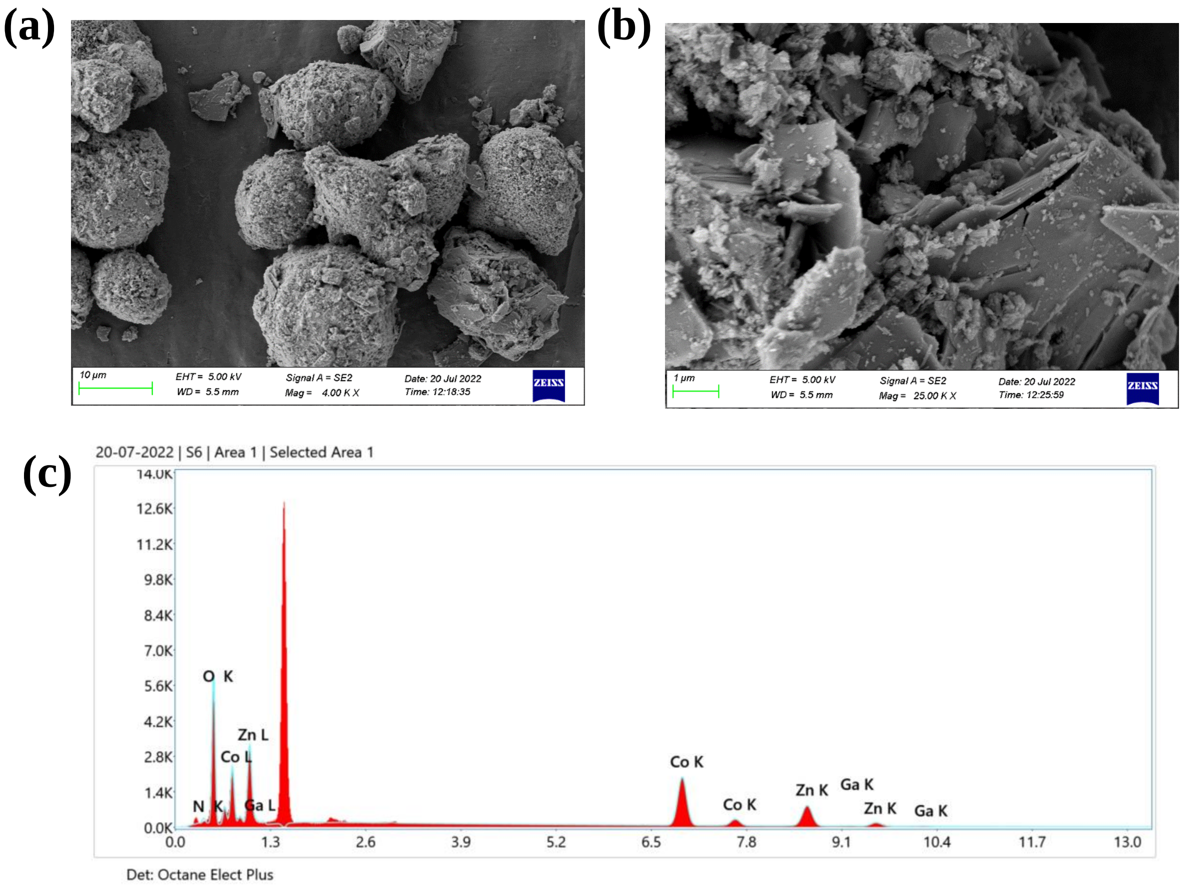

Fig. S20 (a, b) FE-SEM image and (c) EDX spectrum after 6000 GCD cycles.

Table S1 Comparison of various ZCO, GaN-based electrodes with our work:

Sample and Synthesis technique	Specific capacitance	Electrolyte	Device architecture and functionality	Energy density and power density	Potential window	Cyclic stability	Reference
$ZnCo_2O_4$ (Sol-gel)	158 $F.g^{-1}$ at 5 $mV.s^{-1}$ (Three electrode)	1 M KOH	-	-	0.9 V	75 % until 1000 cycles	[3]
GaN (Electrochemical etching)	23.67 $mF.cm^{-2}$ at 0.01 $V.s^{-1}$ (Three electrode) 20.04 $mF.cm^{-2}$ at 0.01 $V.s^{-1}$ (Two electrode)	1 M H_2SO_4	Symmetric supercapacitor	0.65 $\mu Wh.cm^{-2}$ and 45 $mW.cm^{-2}$	0.9 V	99 % until 50000 cycles	[4]
Gallium Oxynitride (Co-precipitation followed by annealing)	217 $F.g^{-1}$ at 0.04 $A.g^{-1}$ (Three electrode)	1 M H_2SO_4	Symmetric supercapacitor	17.2 $\mu Wh.cm^{-2}$ and 0.25 $mW.cm^{-2}$	1.0 V	Up to 10000 cycles	[5]
GaN Nanowire on Graphite paper (Chemical vapor	237 $mF.cm^{-2}$ at 0.1 $mA.cm^{-2}$ (Three electrode)	1 M H_2SO_4	Symmetric supercapacitor	0.30 $mW.h.cm^{-3}$ and 1000 $mW.cm^{-3}$	1.0 V	98 % capacitance retention after 10 000 cycles	[6]

deposition i.e., CVD)							
$ZnCo_2O_4$ @C (Electro-spinning)	90 $F.g^{-1}$ at 8 $A.g^{-1}$ (Three electrode)	6 M KOH	-	-	0.4 V	125 % capacitance retention after 1000 cycles	[7]
Au@rGO@ $ZnCo_2O_4$ (Hydrothermal)	288.5 $mAh.g^{-1}$ at 2 $mV.s^{-1}$ (Three electrode)	KOH	Asymmetric supercapacitor	31 $Wh.kg^{-1}$ and 2121 $W.kg^{-1}$	1.4 V	Up to 5000 cycles	[8]
Activated C_{60}@ $ZnCo_2O_4$ (Solvothermal)	593.2 $F.g^{-1}$ at 1 $mV.s^{-1}$ (Three electrode) 45.54 $F.g^{-1}$ at 2 $A.g^{-1}$ (Two electrode)	2 M KOH	Flexible Asymmetric supercapacitor device	36.43 $Wh.kg^{-1}$ and 1681.47 $W.kg^{-1}$	1.6 V	91.06 % till 6000 cycles	[1]
$ZnCo_2O_4$@MnO_2 core-shell nanosheet arrays on Ni foam (Hydrothermal)	2.60 $F.cm^{-2}$ at a current density of 3 $mA.cm^{-2}$ (Three electrode)	2 M KOH	Asymmetric supercapacitor device	29.41 $Wh.kg^{-1}$ at 628.42 $W.kg^{-1}$	1.55 V	95.3 % till 3000 cycles	[9]

$ZnCo_2O_4$@$ZnWO_4$ nanowire arrays on Ni foam (Hydrothermal)	13.4 $F.cm^{-2}$ at a current density of 4 $mA.cm^{-2}$ (Three electrode)	3 M KOH	Asymmetric supercapacitor device	24 $Wh.kg^{-1}$ at 400 $W.kg^{-1}$	1.6 V	98.5 % retention after 5000 cycles	[10]
rGO@GaN (Chemical reduction process)	454 $F.g^{-1}$ at 10 $mV.s^{-1}$ (Three electrode)	1 M H_2SO_4	-	-	1.45 V	75 % retention after 950 cycles	[11]
GaN/MnO_2/MnON (CVD + Hydrothermal)	532.1 $mAh.cm^{-2}$ at 0.1 $mA.cm^{-2}$ (Three electrode) 438.5 mF cm^{-2} at 0.1 $mA.cm^{-2}$ (Two electrode)	6 M KOH	Flexible symmetric supercapacitor device	0.76 $mW.h.cm^{-3}$ at 125 $mW.cm^{-3}$	1.0 V	95.5 % after 10 000 cycles	[12]
$ZnCo_2O_4$/rGO/NiO on Ni foam (Hydrothermal)	1,256 $F.g^{-1}$ at 3 $A.g^{-1}$ (Three electrode)	6 M KOH	-	62.8 $Wh.kg^{-1}$ at 895 $W.kg^{-1}$	0.6 V	80 % capacitance retention after 3,000 cycles	[13]
$ZnCo_2O_4$@PANI core/shell Nanobelts	1938.2 $F.g^{-1}$ at 1 $A.g^{-1}$ (Three electrode)	2 M KOH	Asymmetric supercapacitor device	66.6 $Wh.kg^{-1}$ at 800.1 $W.kg^{-1}$	1.6 V	92.4% after cycling for 10	[14]

(Hydrothermal)						000 cycles	
$ZnCo_2O_4/ZnO$ nanobelts (Co-precipitation followed by calcination)	481.0 $F.g^{-1}$ at 1 $A.g^{-1}$ (Three electrode)<br>66.9 $F.g^{-1}$ at 1 $A.g^{-1}$ (Two electrode)	3 M KOH	Asymmetric supercapacitor device	23.77 $Wh.kg^{-1}$ at 399.98 $W.kg^{-1}$	1.6 V	67 % maintained after 6000 cycles at 2 $A.g^{-1}$	[15]
**G-ZCO (Hydrothermal)**	**1693 $F.g^{-1}$ at 5 $mV.s^{-1}$ (3 electrode)**<br>**176 $F.g^{-1}$ in 1 $A.g^{-1}$ (2 electrode)**	**2 M KOH + 0.1 M KFeCN (3 electrode)**<br>**2M KOH + 0.1 M KFeCN + PVA gel electrolyte (2 electrode)**	**Ultra-flexible and bendable all-solid-state asymmetric supercapacitor device**	**92.63 $Wh.kg^{-1}$ at 1287.52 $W.kg^{-1}$**	**1.6 V**	**96.7 % retention up to 10000 cycles**	**This work**

Reference:

1 R. Mohanty, G. Swain, K. Parida and K. Parida, *J. Alloys Compd.*, 2022, **919**, 165753.

2 H. Yu, J. Wu, L. Fan, K. Xu, X. Zhong, Y. Lin and J. Lin, *Electrochim. Acta*, 2011, **56**, 6881–6886.

3 L. Merabet, K. Rida and N. Boukmouche, *Ceram. Int.*, 2018, **44**, 11265–11273.

4 S. Wang, L. Zhang, C. Sun, Y. Shao, Y. Wu, J. Lv and X. Hao, *Adv. Mater.*, 2016, **28**, 3768–3776.

5 J. Wang, X. Wu, X. Lu, Z. Xu, H. Jiang, L. Liu, Q. Ban and L. Gai, *Electrochim. Acta*, 2022, **404**, 139733.

6 S. Wang, C. Sun, Y. Shao, Y. Wu, L. Zhang and X. Hao, *Small*, 2017, **13**, 1603330.
7 H. Yu, H. Zhao, Y. Wu, B. Chen and J. Sun, *J. Phys. Chem. Solids*, 2020, **140**, 109385.
8 S. J. Patil, D. P. Dubal and D.-W. Lee, *Chem. Eng. J.*, 2020, **379**, 122211.
9 D. Yu, Z. Zhang, Y. Meng, Y. Teng, Y. Wu, X. Zhang, Q. Sun, W. Tong, X. Zhao and X. Liu, *Inorg. Chem. Front.*, 2018, **5**, 597–604.
10 L. Xie, Y. Liu, H. Bai, C. Li, B. Mao, L. Sun and W. Shi, *J. Colloid Interface Sci.*, 2018, **531**, 64–73.
11 S. Nongthombam, N. A. Devi, S. Sinha, R. Bhujel, S. Rai, W. Ishwarchand, S. Laha and B. P. Swain, *J. Phys. Chem. Solids*, 2020, **141**, 109406.
12 S. Wang, Y. Shao, W. Liu, Y. Wu and X. Hao, *J. Mater. Chem. A*, 2018, **6**, 13215–13224.
13 S. Sahoo and J.-J. Shim, *ACS Sustain. Chem. Eng.*, 2017, **5**, 241–251.
14 X. Chen and J. Cai, *Dalt. Trans.*, 2022, **51**, 16587–16595.
15 Q. Ma, F. Cui, J. Zhang and T. Cui, *J. Colloid Interface Sci.*, 2023, **629**, 649–659.
